# Identification and validation of biomarkers associated with mitochondrial dynamics dysfunction in Parkinson’s disease based on GEO datasets

**DOI:** 10.1371/journal.pone.0362081

**Published:** 2026-11-04

**Authors:** Dawei Gong, Guanyu Li, Ming Li, Bo Zhang, Weixiao Wang, Jinjin Chen, Yanyan Zhao

**Affiliations:** 1 Department of Neurosurgery, The Second Hospital of Nanjing, Affiliated to Nanjing University of Chinese Medicine, Nanjing, China; 2 Department of Neurology, The Second Hospital of Nanjing, Affiliated to Nanjing University of Chinese Medicine, Nanjing, China; 3 Clinical Research Center, The Second Hospital of Nanjing, Affiliated to Nanjing University of Chinese Medicine, Nanjing, China; 4 Department of Neurology, Nanjing BenQ Medical Center, The Affiliated BenQ Hospital of Nanjing Medical University, Nanjing, China; Institut d'Investigacions Biomediques de Barcelona, SPAIN

## Abstract

**Background:**

Parkinson’s disease (PD), the second most prevalent neurodegenerative disorder, is characterized by an unclear etiology and a lack of specific early diagnostic biomarkers. This study investigated novel biomarkers associated with mitochondrial dynamics (MD) dysfunction in PD.

**Methods:**

Transcriptomic data from the Gene Expression Omnibus database (training set: GSE6613, n = 72; validation set: GSE99039, n = 438) and 23 MD-related genes (MDRGs) were integrated. Differentially expressed genes were screened using the limma package. Three machine learning algorithms (random forest, support vector machine, and generalized linear model) were employed to construct diagnostic models. Feature importance analysis identified key candidate biomarkers, which were preliminarily validated in clinical samples via reverse transcription quantitative PCR (RT-qPCR). Functional enrichment, immune infiltration, and compound prediction analyses were subsequently performed.

**Results:**

Cysteinyl leukotriene receptor 2 (*CYSLTR2*) and alpha‑tocopherol transfer protein‑like gene (*TTPAL*) were identified as MDRG score-correlated biomarkers. In the validation set, *CYSLTR2* was significantly downregulated (log_2_FC = −1.25, P < 0.001) and participated in neuroactive ligand–receptor interactions. Conversely, *TTPAL* was significantly upregulated (log_2_FC = 1.52, P < 0.001) and associated with the pathogenic *Escherichia coli* infection pathway. Immune analysis revealed six differentially infiltrated immune cell types. Notably, *TTPAL* expression was positively correlated with keratinocytes (cor = 0.30, P = 0.031). Four potential therapeutic compounds were predicted. RT-qPCR suggested the downregulation of *CYSLTR2* and upregulation of *TTPAL* in patients with PD (P < 0.0001).

**Conclusion:**

This study identified *CYSLTR2* and *TTPAL* as novel candidate biomarkers associated with MD dysfunction in PD, providing novel targets for mechanistic investigation and therapeutic exploration.

## 1 Introduction

Parkinson’s disease (PD) is the second most common neurodegenerative disease after Alzheimer’s disease, and its global burden increases markedly with population aging [1]. PD is predominantly characterized by motor symptoms, including bradykinesia, resting tremor, muscular rigidity, postural instability, and gait disturbances. These debilitating symptoms markedly impair patients’ quality of life and constitute the core clinical diagnostic criteria for PD. Non-motor symptoms, such as olfactory dysfunction and constipation, frequently precede motor symptoms and may represent early indicators of PD, even in its preclinical stage [2,3]. Additionally, the etiology of PD is complex and multifactorial. Accumulating evidence indicates that PD pathogenesis is driven by numerous interwoven contributors, such as genetic predisposition, environmental exposure, and aging-related factors [4]. Moreover, the role of neuroinflammation in PD progression has gained increasing attention [5].

Mitochondria are widely recognized as the “energy factories” of cells and are indispensable for neuronal function and survival. Mitochondrial dynamics (MD) encompass processes including fusion, fission, and mitochondrial transport, which cooperate with biogenesis and mitophagy to maintain mitochondrial turnover. Dysregulated MD can lead to structural and functional abnormalities of mitochondria, which in turn shift the cellular state toward a disease-prone phenotype [6]. Consequently, MD has emerged as a cornerstone of mitochondrial quality control. Accumulating evidence substantiates the pivotal role of MD in the pathogenesis of neurological disorders, such as Alzheimer’s disease, Huntington disease, and PD [7]. Notably, fragmentation and functional impairment driven by dysregulated mitochondrial dynamics hinder mitochondrial recognition and clearance by the autophagic machinery, thereby exacerbating mitophagy defects. This mitophagic impairment has been demonstrated to play a major role in the loss of dopaminergic neurons in PD [8], leading to the widespread acknowledgment that mitochondrial dysfunction is a key pathological marker of PD [9]. Nevertheless, the precise mechanistic underpinnings of MD dysfunction in PD initiation and progression remain to be elucidated.

Owing to the complexity of its etiology and pathogenesis, the current clinical diagnosis of PD primarily relies on clinical manifestations. Among available imaging approaches, [^18^F]DOPA positron emission tomography (PET) can characterize diminished striatal dopaminergic function [10]. Nevertheless, the technical complexity and high cost of PET preclude its application in population-wide early screening [11]. However, by the time typical motor manifestations emerge and a definitive PD diagnosis is established, available therapies can relieve symptoms but not halt or reverse disease progression. Therefore, molecular biomarkers for the early identification of PD are urgently needed to facilitate timely intervention before overt motor manifestations appear. Notably, high-throughput and multi-omic technologies have advanced precision medicine and mechanism-driven drug discovery for neurodegenerative diseases through genome-level targeting of core pathological cascades, characterization of mitochondrial molecular disorders, and improved molecular diagnosis of mitochondrial-related diseases [12–14]. Via integrated analysis of the Gene Expression Omnibus (GEO) datasets and MD-related genes (MDRGs), this study aims to identify novel biomarkers associated with MD dysfunction in PD and systematically elucidate their potential regulatory mechanisms. These findings will offer new insights into the pathological mechanisms underlying PD and highlight novel targets for mechanistic investigation and therapeutic exploration.

## 2. Materials and methods

### 2.1. Data collection

Transcriptomic datasets were retrieved from the GEO database (https://www.ncbi.nlm.nih.gov/geo/) on June 15, 2025. All datasets used in this study were fully anonymized, and no personally identifiable information of individual participants was accessible to the authors. The GSE6613 dataset was used as the training set for biomarker screening. The sequencing platform was GPL96, and this dataset contained whole-blood samples from 50 patients with PD and 22 healthy controls. The GSE99039 dataset was used as completely independent external validation cohort to assess the generalizability of findings. The sequencing platform was GPL570, and this dataset contained peripheral blood samples from 205 patients with PD and 233 healthy controls. Twenty-three MDRGs were obtained from the MitoCarta 3.0 database [15]: *ARMC10*, *ARL2*, *DNM1L* (also termed *Drp1*), *MFN1*, *FIS1*, *MFN2*, *MFF*, *MIGA1*, *MIEF1*, *MIGA2*, *MIEF2*, *MTCH2*, *MTFP1*, *MTFR1*, *OPA1*, *MTFR2*, *PLD6*, *MUL1*, *OMA1*, *RAB24*, *SLC25A46*, *SPIRE1*, and *STX17*.

### 2.2 Identification of candidate genes

The limma package (v3.44.3) [16] was employed to perform differential expression analysis between PD and control samples in the training set to obtain differentially expressed genes (DEGs). DEGs were filtered according to the thresholds of P < 0.05 and |log_2_FC| > 0.5. Additionally, the ggplot2 (v3.3.2) [17] and ComplexHeatmap [18] packages were employed to visualize the volcano map and heatmap of DEGs, respectively. Subsequently, based on the MDRG list, the transcriptomic matrix of the GSE6613 dataset was converted into a score matrix using the gene set variation analysis (GSVA) algorithm. Differences in GSVA scores between PD patients and healthy controls were assessed using the Wilcoxon rank-sum test. PD samples were stratified into high- and low-score groups based on the median GSVA score. Thresholds of P < 0.05 and |log_2_FC| > 0.5 were used to screen DEGs associated with MDRGs. Finally, the DEGs between PD patients and healthy controls and those associated with MDRGs were intersected to obtain candidate genes.

### 2.3 Enrichment analysis of candidate genes and construction of the protein–protein interaction (PPI) network

To investigate the potential functional pathways of candidate genes, these genes were subjected to Gene Ontology (GO) and Kyoto Encyclopedia of Genes and Genomes (KEGG) enrichment analyses using the clusterProfiler package (v3.16.0), with a threshold of P < 0.05 [19,20]. The top 15 significantly enriched pathways were obtained. To further investigate the interactions among candidate genes at the protein level, a PPI network (combined score > 0.4) was constructed using the STRING database (http://string-db.org/) [21] and visualized using Cytoscape (v3.9.1) [22].

### 2.4 Machine learning screening for biomarkers

Prior to machine learning modeling, uniform preprocessing was performed on raw gene expression data, including missing value removal, quantile normalization, and batch effect correction to eliminate nonbiological experimental noise. In the present study, the GSE6613 dataset was used as the training cohort for model construction, while the independent dataset GSE99039 served as the external validation cohort. Given the relatively limited sample size of the training set, five-fold stratified cross-validation was implemented for all classification models to balance the proportions of PD patient and healthy control samples and minimize bias attributable to random grouping. Hyperparameter tuning for all models was completed via the built-in grid search algorithm of the caret package (v6.0.49) [23], and regularization constraints were incorporated to reduce model complexity and substantially mitigate overfitting.

Random forest (RF), support vector machine (SVM), and generalized linear model (GLM) were employed using the caret package for the first‑round modeling. Preliminary candidate genes were set as explanatory variables, while disease status (PD versus healthy control) was defined as the response variable.

The explain function of the DALEX package (v2.4.3) [24] was used to generate explainer objects for the three models. Residual distributions were calculated from observed and predicted values, and permutation-based variable importance was used to quantify the contribution of each explanatory variable. For consistent comparison, the same residual definition and evaluation framework were applied to RF, SVM, and GLM. The RF model with the lowest overall residual error was selected as the optimal model. Permutation-based variable importance scores were computed for all three models via DALEX, and scores derived from the optimal RF model were adopted to select the top 10 genes as candidate biomarkers.

To verify whether the expression trends of the candidate biomarkers were consistent between the training and validation sets, the limma package (v3.44.3) was used to analyze biomarker expression (P < 0.05). Only candidate biomarkers exhibiting consistent and statistically significant expression trends in both datasets were retained as identified biomarkers.

Having obtained the two final identified biomarkers, we performed second‑round modeling to evaluate their disease‑status classification ability by constructing three machine‑learning classifiers (RF, GLM, and SVM) using their expression matrix. Disease status (control/disease) was used as the classification label, while the expression levels of these two identified biomarkers were used as input features. The three models were trained separately using the R package caret. Subsequently, receiver operating characteristic (ROC) curves were used to evaluate the classification performance of the different machine-learning models on the training dataset. The area under the curve (AUC) and its 95% confidence interval (95% CI) were calculated using the pROC package to compare the discriminatory ability of the different algorithms between disease and control samples. Furthermore, 5‑fold stratified cross‑validation was performed within the GSE6613 training cohort in this second‑round modeling as an additional analysis to maintain balanced PD‑control sample ratios across folds and reduce bias from single data splitting. Specifically, the cross-validation framework implemented in the R package caret was used to retrain and evaluate the RF, SVM, and GLM models. During cross-validation, model-specific parameter tuning was performed within the training folds to determine the corresponding model parameters and reduce the variability associated with a single model fit. After model construction and internal five‑fold stratified cross‑validation within GSE6613, the trained RF, SVM and GLM classification models were directly applied to the fully independent external validation cohort GSE99039. Receiver‑operating characteristic curve analysis was performed to calculate AUC values for evaluating the generalizability of these machine‑learning classifiers.

### 2.5 Nomogram construction and assessment of biomarker performance

To further evaluate the predictive performance of the biomarkers for PD, receiver operating characteristic (ROC) curves were plotted for both the training and validation sets using the pROC package (with an AUC greater than 0.7 indicating good predictive accuracy). Meanwhile, to assess the diagnostic value of these biomarkers for PD, a scoring system was constructed based on the biomarker expression levels in the training set. Each biomarker was assigned an individual score, and the sum of these individual scores yielded the total score, which was subsequently used to predict the diagnostic probability of PD. Higher total scores corresponded to an increased probability of PD diagnosis. A biomarker-based nomogram was constructed using the rms package. The predictive performance of the nomogram was comprehensively assessed via calibration curves, ROC curve analysis, and decision curve analysis (DCA).

### 2.6 Enrichment analysis of biomarkers

The potential pathways of the biomarkers were investigated. For each biomarker, Spearman’s correlation coefficients were calculated between the biomarker and all other genes across samples. Genes were ranked in descending order of correlation coefficients to generate pre-ranked gene lists. Subsequently, gene set enrichment analysis (GSEA) [25] was performed using the clusterProfiler package (v4.8.3) [26], with KEGG gene sets as the reference gene collection [20]. The number of permutations was set to 10,000, and the Benjamini–Hochberg procedure was applied for correction. Pathways with FDR-adjusted P < 0.05 were defined as significantly enriched (|normalized enrichment score| > 1). The leading-edge genes of all significantly enriched pathways were extracted simultaneously.

### 2.7 Immunocyte infiltration analysis

To evaluate immune cell infiltration in PD, infiltration scores of 64 immune cell types in the training set were calculated using the xCell algorithm (v1.1.0) [27]. The Wilcoxon rank-sum test was employed to identify the immune cells with differential infiltration between PD and control samples (P < 0.05), and the results were visualized as a box plot using the ggplot2 package (v3.3.2). After identifying differentially infiltrated immune cell subtypes, Spearman’s correlation was analyzed using the stats package and visualized using the corrplot package (v0.92) [28], with predefined screening thresholds of |cor| > 0.3 and P < 0.05.

### 2.8 Regulatory networks of biomarkers and transcription factors (TFs)

We retrieved potential transcription factors (TFs) and microRNAs (miRNAs) targeting biomarkers through the NetworkAnalyst platform (https://www.networkanalyst.ca/). TF–gene binding profiles were derived from the JASPAR database, and miRNA–gene interaction data were extracted from the miRTarBase database. The TF–biomarker regulatory network and the integrated TF–miRNA–biomarker regulatory network were visualized and refined using Cytoscape software (version 3.9.1).

### 2.9 Compound prediction and molecular docking

To screen potential compounds for PD treatment, the Comparative Toxicogenomics Database (https://ctdbase.org/) was employed to predict small-molecule compounds acting on the biomarkers. The top 20 compounds were retained for further analysis, and the biomarker–compound interaction network was constructed using Cytoscape (v3.9.1) to identify potential therapeutic compounds for PD. Subsequently, molecular docking was conducted via CB-Dock2 (https://cadd.labshare.cn/cb-dock2/php/index.php) [29] to evaluate binding affinities between the compounds and their target biomarker proteins. Three-dimensional structures of the compounds were retrieved from the PubChem database (https://pubchem.ncbi.nlm.nih.gov/), while high-resolution receptor structures of the biomarker proteins were obtained from the Protein Data Bank (PDB, https://www.rcsb.org/). CurPocket was used to predict the top five potential binding pockets, with AutoDock Vina serving as the underlying docking engine. Heteroatoms and water molecules were removed from receptor structures, and grid boxes were defined based on the geometric boundaries of predicted cavities. Docking calculations were repeated multiple times, and conformations with root‑mean‑square deviation < 5 Å were retained. For benchmarking purposes, the endogenous CYSLTR2 ligands leukotriene C4 (LTC4), leukotriene D4 (LTD4), and leukotriene E4 (LTE4) were docked under identical computational parameters, using the same receptor structure, pocket prediction, and calculation workflow. For all docking poses derived from both candidate compounds and endogenous ligands, key interacting residues and ligand–protein contact distances were analyzed and quantified. All docking outcomes were ranked according to Vina binding scores and visualized using PyMOL software.

### 2.10 Subcellular localization and gene–gene interaction (GGI) network analysis

To characterize the biological functions of proteins encoded by the candidate biomarkers, we explored their subcellular locations to identify their functional compartments. Subcellular localization information of biomarkers was retrieved from the GeneCards database (https://www.genecards.org/). In addition, GeneMANIA (https://genemania.org/) was used to collect genes with similar functional profiles to the target biomarkers, and a gene–gene interaction (GGI) network was built from these functional associations.

### 2.11 Validation of biomarker expression

Reverse transcription quantitative PCR (RT-qPCR) was employed to confirm the consistency of candidate biomarker expression with the bioinformatic results. This study was approved by the Ethics Committee of the Second Hospital of Nanjing (approval No.: 2025-LS-ky066, date: June 10, 2025). Clinical samples were obtained for research purposes on August 25, 2025. No personally identifiable information of participants was accessible during and after data collection. All samples were collected from the Second Hospital of Nanjing, and all donors provided written informed consent. Specifically, whole-blood samples were collected from five patients with PD and five healthy individuals. Total RNA was extracted from each sample using the TRIzol reagent (Cat. No. 15596018CN, Thermo Fisher Scientific, Waltham, MA, USA), and cDNA was synthesized using the SweScript First Strand cDNA Synthesis Kit (Cat. No. G3333-50, Servicebio, Wuhan, China). *GAPDH* was employed as an internal reference gene, and the 2^−ΔΔCt^ method [30] was used to calculate candidate biomarker expression. Primer sequences are listed in Table 1. The resultant data were statistically analyzed and visualized using GraphPad Prism (v8.0).

**Table 1:** Primer sequences for quantitative real‑time PCR.

Genes	Forward primer	Reverse primer
*CYSLTR2*	TGCAAGGTATGGAGAGTTCCTCAA	TGAAGAGGTGATCCCCAGGAA
*TTPAL*	GCCGGGACCTTGGTAGCTAAT	CGAAGTCTCCATTCCGGCTT
*GAPDH*	ATGGGCAGCCGTTAGGAAAG	AGGAAAAGCATCACCCGGAG

Note: *GAPDH* was used as the internal reference gene. *CYSLTR2*, cysteinyl leukotriene receptor 2; *TTPAL*, alpha‑tocopherol transfer protein‑like.

### 2.12 Statistical analysis

Bioinformatic analyses were performed using R (v4.2.2). The Wilcoxon rank-sum test and Student’s *t*-test were used for comparisons between two groups. Fisher’s exact test was applied to calculate the significance of enriched terms in GO and KEGG pathway analyses, and *P* < 0.05 was defined as the threshold for statistically significant results.

### 2.13 Ethical approval and consent to participate

This study was performed in line with the principles of the Declaration of Helsinki. All participants provided written informed consent prior to enrollment. The relevant ethical approval information has been detailed in Section 2.11.

## 3 Results

### 3.1 Identification of candidate genes related to MD in PD

In total, 221 DEGs were obtained from the GSE6613 dataset using the predefined screening thresholds, consisting of 121 upregulated and 100 downregulated genes (Fig 1A). Distinct expression patterns of the top 20 DEGs were observed between PD and control samples (Fig 1B). The MDRG scores of PD samples were lower than those of control samples (Fig 1C). Subsequently, PD samples were stratified into high- and low-score groups based on the median MDRG score (−0.08153), with 25 PD samples in each group. After screening using the predefined thresholds, 381 DEGs associated with MDRGs were obtained between the high- and low-score groups (Fig 1D). Similarly, the upregulated and downregulated MDRGs also exhibited different expression profiles (Fig 1E). Finally, the 221 DEGs and 381 MDRG-related DEGs derived from the training set were intersected, yielding 17 candidate genes (Fig 1F).

**Fig 1:**
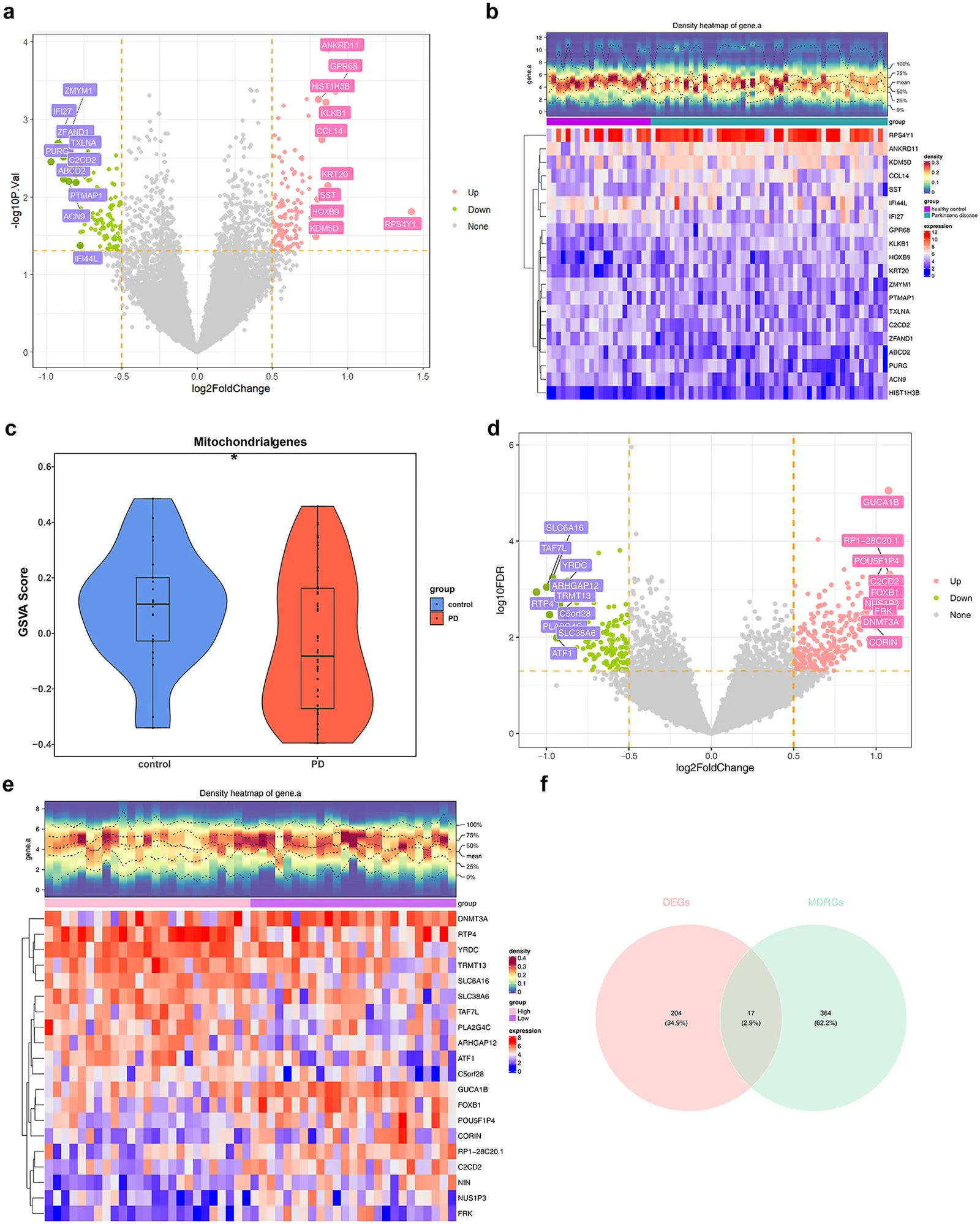
Identification of candidate genes related to MD in PD. (A) Volcano plot showing differentially expressed genes (DEGs) between PD and control samples. (B) Heatmap of DEGs between PD and control samples. (C) Comparison of MDRG scores between PD and control samples. (D) Volcano plot of differential genes between the high- and low-score groups. (E) Heatmap of differential genes between the high- and low-score groups. (F) Venn diagram for identifying candidate genes.

### 3.2 Functional and interactive characterization of candidate genes

Potential functional pathways associated with the candidate genes were explored via GO and KEGG enrichment analyses. In the GO enrichment results, candidate genes were mainly enriched in transcription elongation by mitochondrial RNA polymerase (GO:0006392), O-acyltransferase activity (GO:0008374), and polymeric immunoglobulin receptor-mediated immunoglobulin transport in epithelial cells (GO:0002415; Fig 2A). In the KEGG enrichment results, candidate genes were mainly enriched in steroid biosynthesis (hsa00100), terpene skeleton biosynthesis (hsa00900), and α-linolenic acid metabolism (hsa00592; Fig 2B). Thus, these candidate genes might be closely correlated with PD pathogenesis and the MD dysfunction-related mechanism. Furthermore, seven proteins encoded by the candidate genes interacted with each other within the PPI network (RAB17, RIPK4, C2CD2, TEFM, TRMT13, PLA2G4C, and SOAT2), and four interaction pairs were identified (Fig 2C).

**Fig 2:**
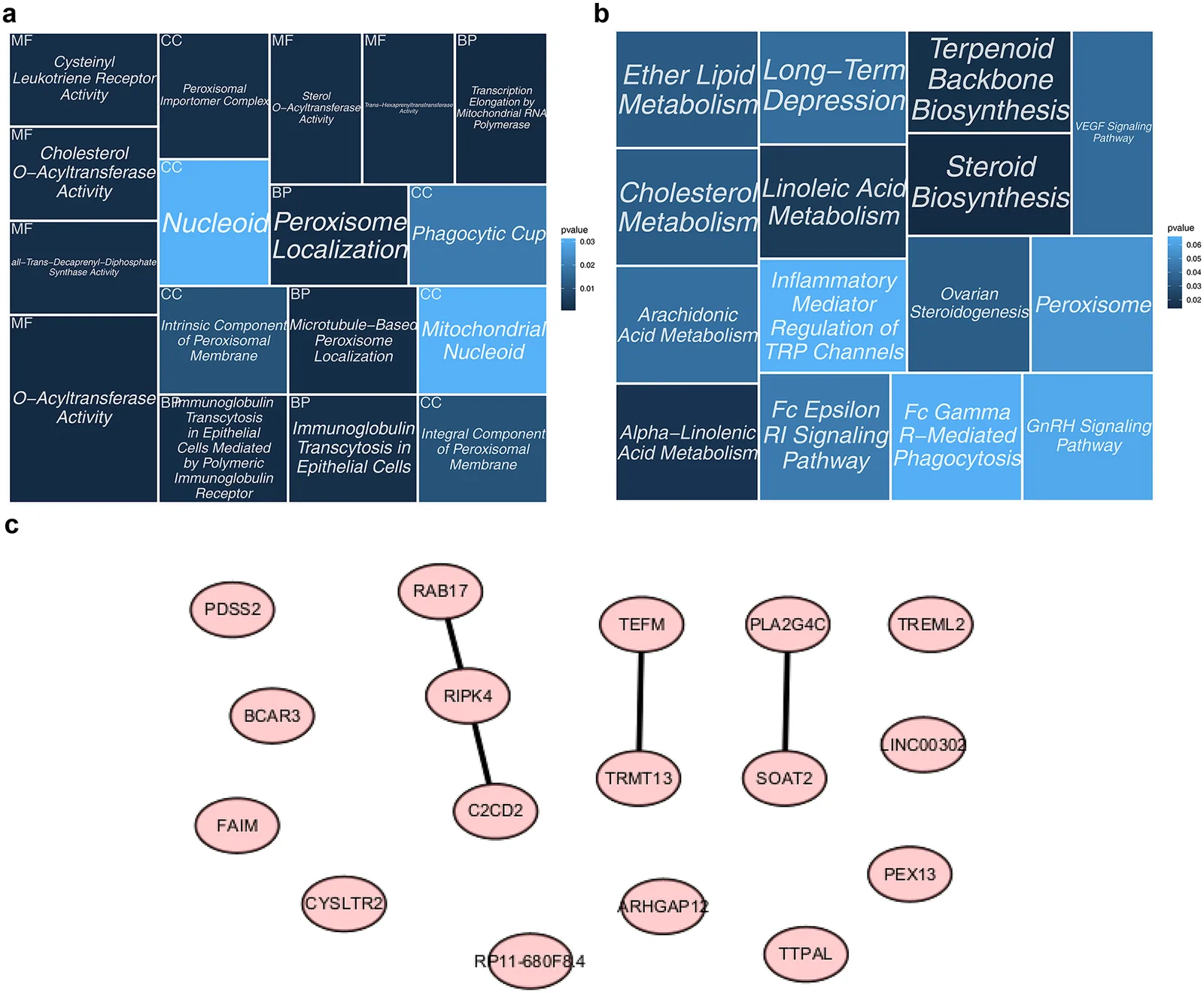
Functional enrichment analysis and PPI network of candidate genes. (A) GO enrichment analysis of candidate genes. (B) KEGG enrichment analysis of candidate genes. (C) PPI network constructed using candidate genes.

### 3.3 MD dysfunction-related biomarkers in PD

Model‑level performance across RF, SVM, and GLM was compared using unified residual metrics implemented in DALEX (Fig 3A). The RF model yielded the smallest model residuals calculated using the DALEX package (evaluated via the reverse cumulative distribution of absolute residuals), and was selected as the optimal model (Fig 3B–C). Permutation‑based variable importance for explanatory variables was calculated for all three models (Fig 3A), and feature importance outputs from the optimal RF model were adopted for biomarker screening. Using these RF‑derived importance scores, the top 10 genes [*PDSS2*, cysteinyl leukotriene receptor 2 (*CYSLTR2*), *LINC00302*, *RAB17*, *RIPK4*, *ARHGAP12*, *C2CD2*, *TRMT13*, *SOAT2*, and alpha‑tocopherol transfer protein‑like (*TTPAL*)] were selected as candidate biomarkers (Fig 3D). Subsequent validation in the training and validation sets (P < 0.05) revealed that *CYSLTR2* was significantly downregulated in PD samples, whereas *TTPAL* was significantly upregulated (Fig 3E–F), establishing these two genes as biomarkers associated with MD dysfunction. In addition, RF, GLM, and SVM classification models were constructed based on the expression profiles of the identified biomarkers, and ROC curve analysis was performed to evaluate their classification performance. The results showed that all three machine-learning models exhibited good discriminatory ability within the training set. Among them, the random forest model achieved the highest classification performance, with an AUC of 1.000 (95% CI: 1.000–1.000). The support vector machine model achieved an AUC of 0.997 (95% CI: 0.991–1.000), while the generalized linear model achieved an AUC of 0.990 (95% CI: 0.975–1.000) (Fig 3G). Furthermore, 5-fold stratified cross-validation was performed to further assess model stability. The results showed that all three models retained good classification performance under 5-fold cross-validation. Specifically, the cross-validated AUC was 0.839 (95% CI: 0.786–0.891) for the RF model, 0.818 (95% CI: 0.713–0.923) for the GLM model, and 0.925 (95% CI: 0.887–0.962) for the SVM model (Fig 3H). These findings indicated that the models constructed using the candidate genes maintained a certain degree of discriminatory ability under cross-validation, lending support to the robustness of our candidate biomarker screening. It should be noted that the near‑perfect apparent AUC values observed in the training set, accompanied by relatively lower cross‑validated AUCs, indicate potential model overfitting. Thus, the predictive performance estimated from the training set should be interpreted with caution. Notably, classification performance dropped substantially in the fully independent external cohort GSE99039, which highlights limitations for the direct clinical diagnostic application of these models.

**Fig 3:**
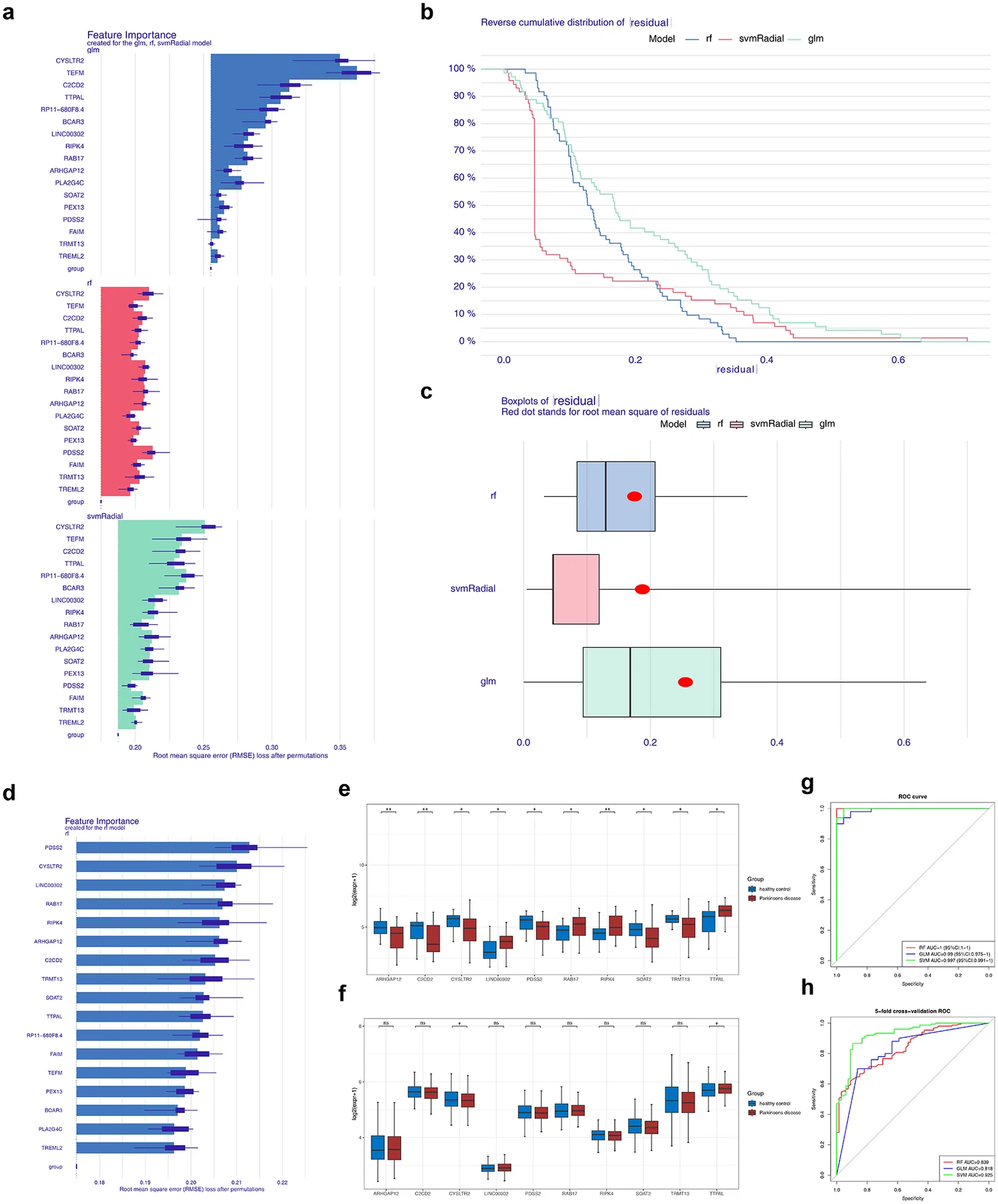
Machine learning-based screening of PD biomarkers related to MD dysfunction. (A) Construction of RF, SVM, and GLM models using candidate genes. (B) Inverse cumulative distribution of residuals for comparative analysis across RF, SVM, and GLM models. (C) Boxplots of residuals showing residual distributions for the RF, SVM, and GLM models. (D) The top 10 genes ranked by permutation‑based feature importance derived from the optimal RF model. (E–F) Boxplots of the expression of the 10 genes in the training and validation sets. (G) ROC curves and AUC values of the RF, GLM, and SVM classification models based on the identified candidate biomarkers. (H) ROC curves and cross-validated AUC values of the RF, GLM, and SVM models under 5-fold stratified cross-validation.

### 3.4 Diagnostic performance of individual genes and combined nomogram model

*CYSLTR2* and *TTPAL* yielded AUCs ranging from 0.549 to 0.667 in the training (S1A Fig) and validation sets (S1B Fig), indicating limited discriminatory performance for either gene alone. A combined predictive model was constructed using *CYSLTR2* and *TTPAL,* and the corresponding nomogram was generated (S1C Fig). The calibration curve revealed that the predicted values closely agreed with the observed values, demonstrating favorable predictive consistency for the model (S1D Fig). Meanwhile, the AUC for the model reached 0.707, indicating that it exhibited reliable diagnostic efficacy (S1E Fig). DCA further confirmed that the model achieved higher clinical net benefit and superior overall predictive performance (S1F Fig).

### 3.5 Functional enrichment and immune microenvironment analysis

GSEA based on the KEGG database illustrated that *CYSLTR2* was significantly enriched in 16 pathways, including the ribosome, spliceosome, and neuroactive ligand–receptor interaction (Fig 4A). TTPAL was involved in the ribosome and pathogenic *Escherichia coli* infection pathways. Specifically, the pathogenic *E. coli* infection pathway was downregulated, and the ribosomal pathway tended to be upregulated (Fig 4B). Thus, these two biomarkers might participate in the onset and development of PD via these important pathways. The infiltration scores of 64 immune cell types were calculated for patients with PD and healthy controls in the training set using the xCell algorithm (v1.1.0; Fig 4C). The results revealed six differential immune cell types (P < 0.05): gamma-delta T (γδ T) cells, natural killer T (NKT) cells, naïve CD8^+^ T cells, keratinocytes, common lymphoid progenitor cells, and CD8^+^ T cells (Fig 4D). The strongest negative correlation was observed between CD8^+^ T cells and NKT cells (cor = −0.334, P = 0.004), while the strongest positive correlation existed between naïve CD8^+^ T cells and CD8^+^ T cells (cor = 0.562, P < 0.0001; Fig 4E). Regarding the interactions between the two biomarkers and immune cell subtypes, the strongest positive correlation was detected between *TTPAL* and keratinocytes (cor = 0.300, P = 0.031; Fig 4F), suggesting that these biomarkers might influence the initiation and progression of PD by remodeling the immune microenvironment.

**Fig 4:**
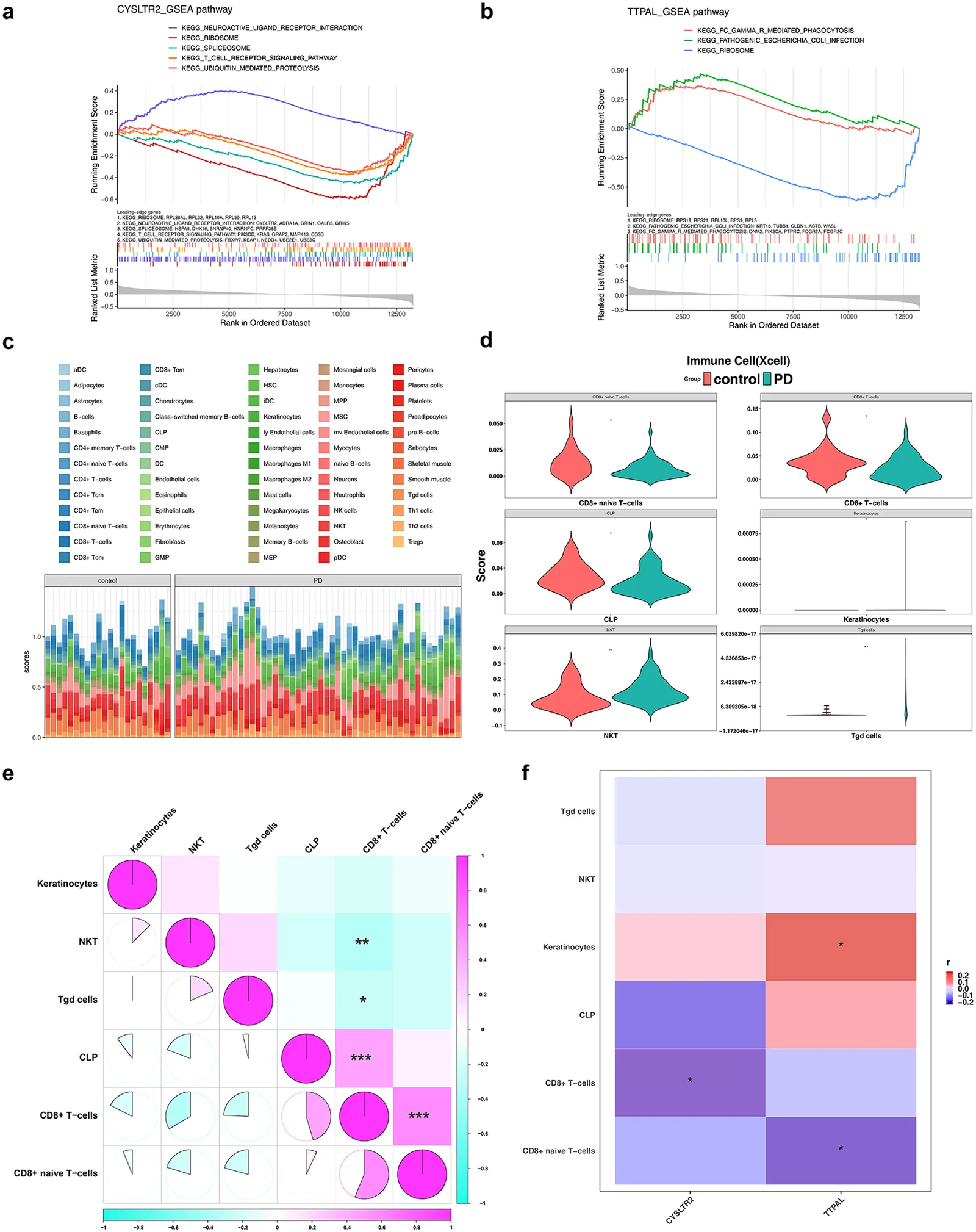
Immune infiltration and functional enrichment analyses. (A) Enrichment analysis of *CYSLTR2*. (B) Enrichment analysis of *TTPAL*. (C) Infiltration scores of 64 immune cell types in PD patients and healthy controls from the training set. (D) Differential immune cells between PD patients and healthy controls in the training set. (E) Correlations heatmap among differential immune cells. (F) Correlations heatmap between differential immune cells and the two biomarkers.

### 3.6 The possibility of targeted biomarker regulation

The results indicated that *CYSLTR2* and *TTPAL* potentially interacted with nine TFs, and both genes were projected to interact with E2F1 and TP53. Subsequently, 22 miRNAs targeting *CYSLTR2* and 58 miRNAs targeting *TTPAL* were identified. Following these analyses, the integrated TF–miRNA–biomarker regulatory network was constructed and contained 98 nodes and 98 edges (Fig 5A). These findings supplied a basis for the targeted regulation of biomarkers.

**Fig 5:**
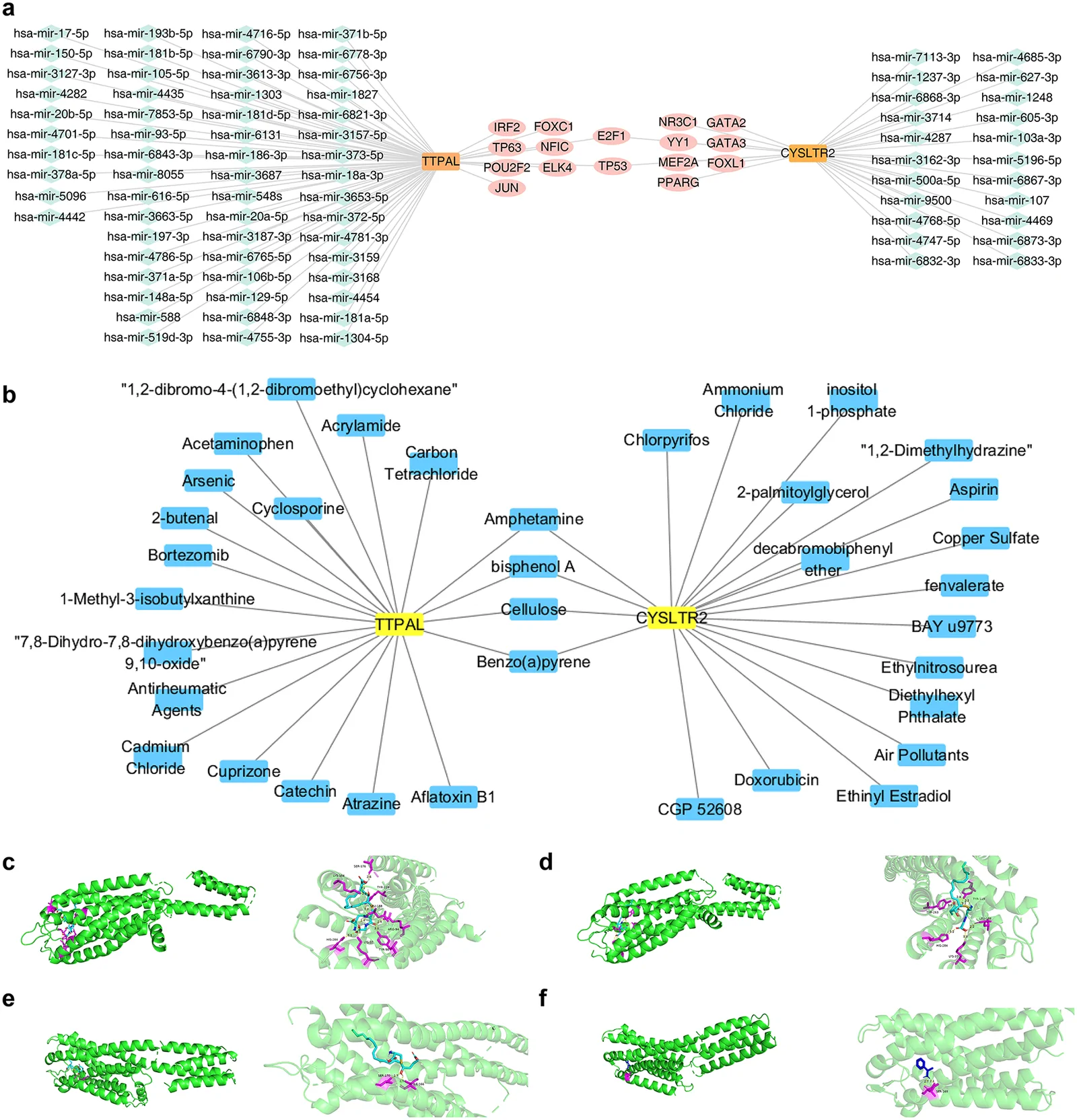
Regulatory network, compound prediction and molecular docking analysis of the identified biomarkers.

### 3.7 Compound prediction and molecular docking of the two biomarkers

To identify potential therapeutic agents for PD, interactions between the biomarkers and available therapeutic agents were explored. The top 20 predicted small-molecule compounds were selected to construct a biomarker–compound interaction network. This network featured 38 nodes and 40 edges, and four compounds [amphetamine, bisphenol A, cellulose, and benzo[a]pyrene] interacted with *CYSLTR2* and *TTPAL* (Fig 5B), thus representing potential therapeutic compounds of interest. Molecular docking was employed to determine the binding of compounds to biomarkers. Among the CTD-predicted compounds, amphetamine was selected for illustrative docking because of its documented effects on striatal dopamine release in PD [31], and the availability of a CYSLTR2 protein structure. Since only the 3D structure of CYSLTR2 was available in the PDB database, CYSLTR2 was employed as the receptor. To compare the predicted binding capacity of amphetamine with that of classical endogenous ligands of CYSLTR2, LTC4 (Fig 5C), LTD4 (Fig 5D), and LTE4 (Fig 5E) were further selected and subjected to molecular docking using the CB-Dock2 platform under the same conditions. The predicted binding energy of amphetamine with CYSLTR2 was −6.6 kcal/mol, whereas those of LTC4, LTD4, and LTE4 were −8.9, −7.7, and −7.8 kcal/mol, respectively, indicating that amphetamine exhibited weaker predicted binding than the endogenous ligands. Further analysis of the binding mode showed that amphetamine could enter the predicted binding pocket of CYSLTR2 and interact with residue SER169 at a distance of 2.6 Å (Fig 5F). The predicted binding energies, key interacting residues, and interaction distances for amphetamine and the three endogenous ligands are summarized in Table 2. These findings suggest a potential structural interaction between amphetamine and CYSLTR2; however, this conclusion is based solely on in silico predictions and requires further experimental validation.

**Table 2:** Molecular docking results of amphetamine and endogenous ligands with CYSLTR2.

Target protein (PDB ID)	Ligand	Binding energy (kcal/mol)	Key interacting residues (contact distance, Å)
CYSLTR2 (6RZ6)	Amphetamine	−6.6	Ser**-**169 (2.6)
	LTC4	−8.9	Lys-194 (2.9,3.1); Ser-176 (2.6); Tyr-119 (3.0); Leu-188 (2.4); Arg-94 (2.8); Tyr-98 (3.0); Lys-37 (3.0); His-284 (3.1)
	
	LTD4	−7.7	Tyr-263 (3.0); Tyr-119 (2.8,3.2,2.1); Leu-188 (2.2); Lys-37 (3.0); His-284 (3.2)
	
	LTE4	−7.8	Ser-170 (2.7); Ile-166 (2.5)
	

Note: Vina scores represent predicted binding affinities; lower values indicate stronger binding. Residues denote key receptor amino‑acid residues interacting with each ligand.

LTC4, leukotriene C4; LTD4, leukotriene D4; LTE4, leukotriene E4.

### 3.8 Subcellular localization and GGI network of *CYSLTR2* and *TTPAL*

The subcellular localization of the proteins encoded by these two biomarkers indicated their potential functions across multiple cellular compartments: *CYSLTR2* was predominantly localized to the plasma membrane (Fig 6A), whereas *TTPAL* was mainly distributed in the cytosol (Fig 6B). To explore functional associations between the two biomarkers, as well as between the biomarkers and their partner genes, GGI networks were constructed for *CYSLTR2* and *TTPAL*. In total, 10 and 11 functionally similar target genes were predicted for *CYSLTR2* and *TTPAL*, respectively. Functional pathways predicted for the biomarkers and their target genes included the dopamine receptor signaling pathway, phosphatidylinositol–phosphate binding, phosphatidylinositol binding, and the GTPase complex (Fig 6C).

**Fig 6:**
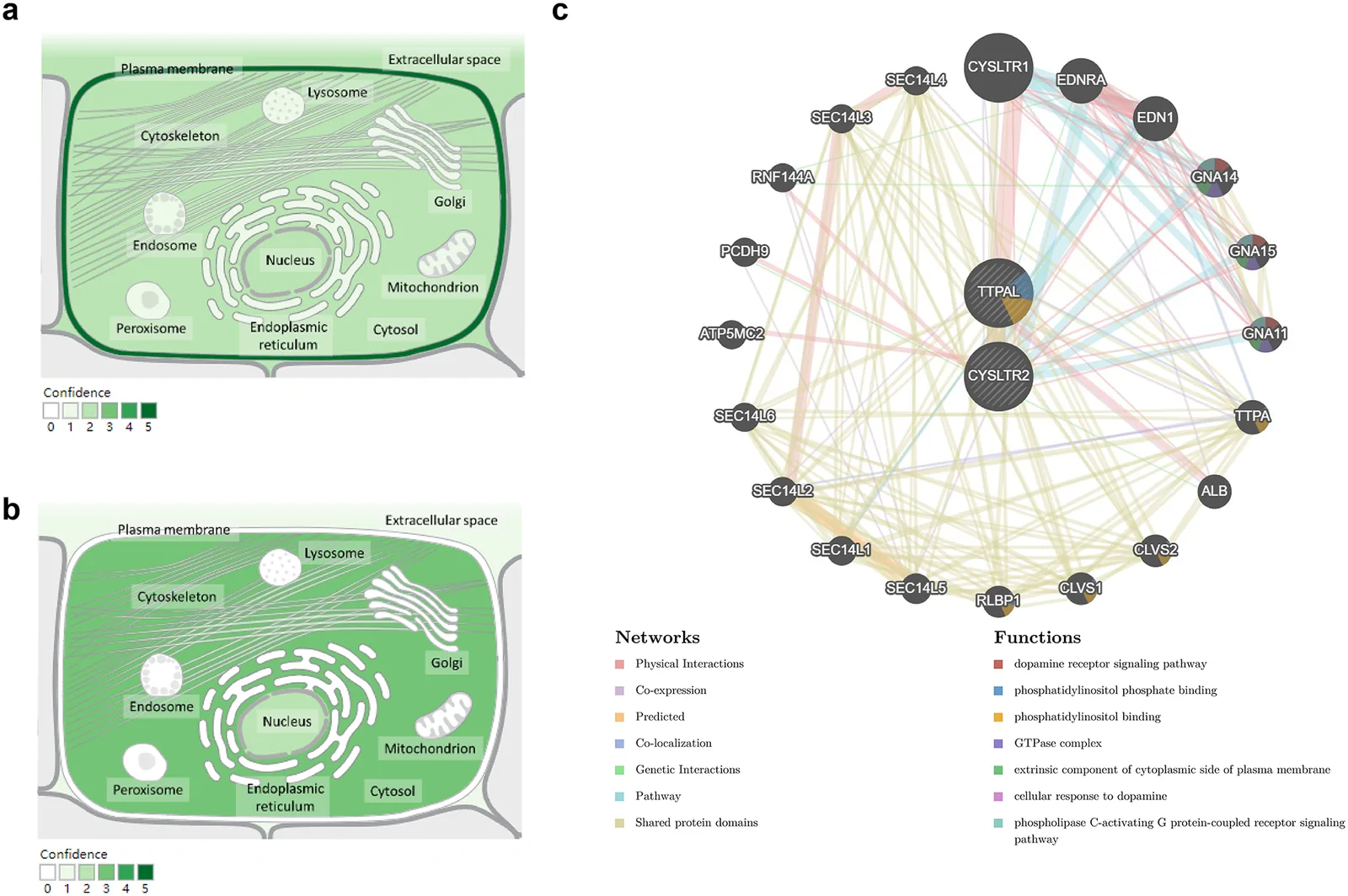
Subcellular localization and GGI network construction for *CYSLTR2* and *TTPAL.* (A) Subcellular localization of *CYSLTR2*. (B) Subcellular localization of *TTPAL*. (C) GGI network constructed for the two biomarkers.

### 3.9 Further verification of the two biomarkers in clinical samples

RT-qPCR results further validated the reliability of these identified biomarkers. Specifically, *CYSLTR2* exhibited reduced expression in the PD group, and *TTPAL* showed elevated expression (both P < 0.0001; Fig 7A‑B), consistent with the expression patterns observed in the GSE6613 and GSE99039 datasets. Given the small sample size, these findings should be interpreted with caution.

**Fig 7:**
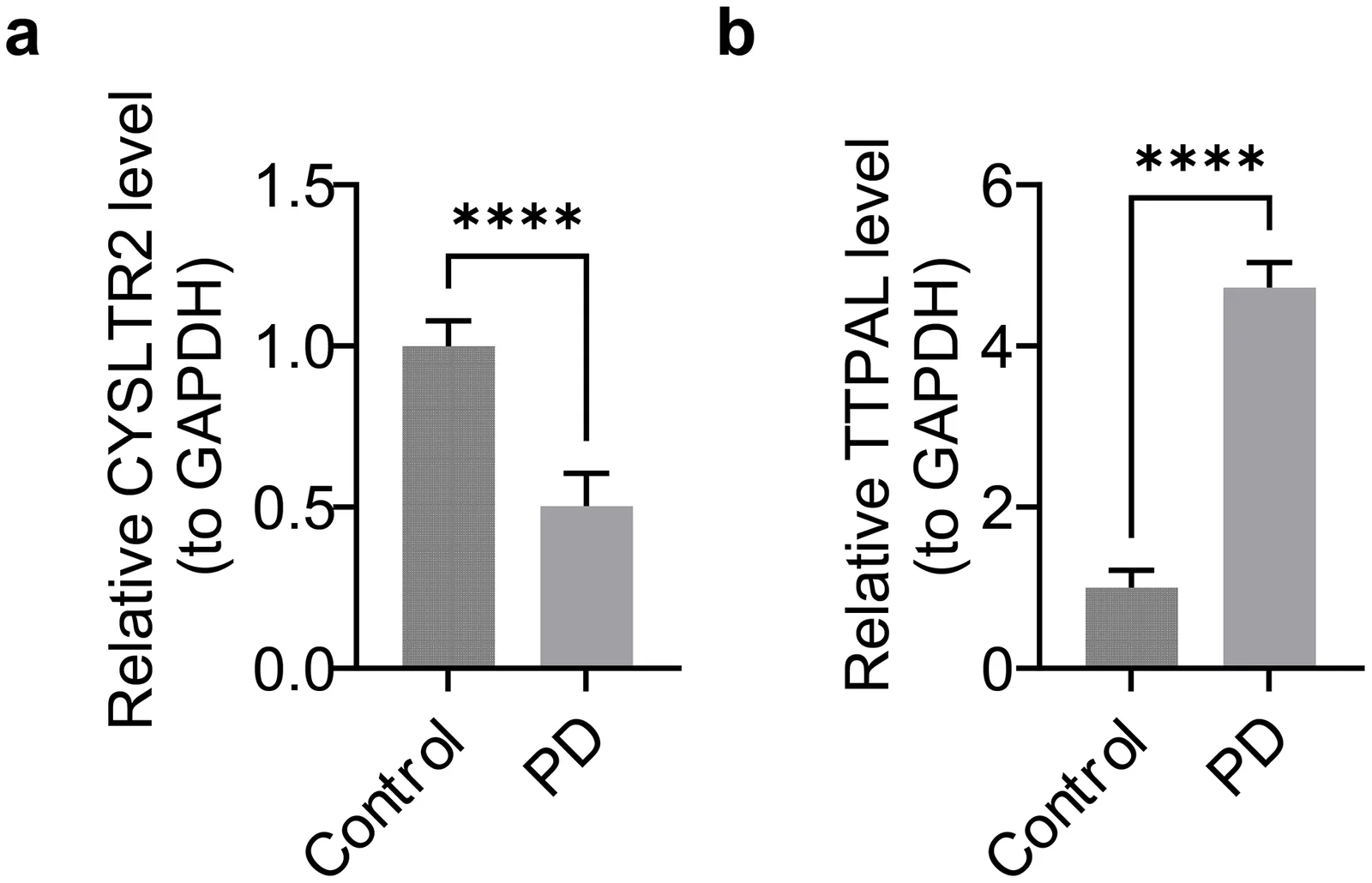
Further verification of biomarkers in clinical samples. (A) *CYSLTR2* expression was significantly downregulated in the PD group. (B) *TTPAL* expression was significantly upregulated in the PD group.

## 4 Discussion

PD is a common age-related neurodegenerative disorder [1]. Mitochondrial dysfunction drives pathological phenotypes in diverse diseases; oncology studies show that disrupted mitochondrial dynamics regulate cell fate, immune function, and disease progression [32,33]. Whereas prior studies have implicated MD in the pathogenesis of neurological diseases [7], the underlying molecular mechanisms driving PD progression remain unclear. Nevertheless, mitochondria-targeted therapies are garnering growing interest in neurodegeneration [34], while some signaling pathways governing mitochondrial homeostasis have emerged as promising potential drug targets for PD [35]. In this study, we integrated GEO transcriptome datasets with established MDRGs. Through comprehensive bioinformatic analyses, we identified two novel biomarkers, elucidated their associated pathways, and provided novel insights into the pathological mechanisms of PD, offering potential targets for mechanistic investigation and therapeutic exploration.

In this study, *CYSLTR2* and *TTPAL* were identified as biologically significant biomarkers whose expression levels exhibited significant correlations with MDRG-derived MD scores in PD. *CYSLTR2* (GeneID: 57105) encodes a G protein-coupled receptor that mediates cellular responses to cysteine leukotrienes and serves as an endogenous ceramide receptor. Ceramides, as bioactive lipids, are involved in cell survival and cellular stress responses. Emerging evidence indicates that *CYSLTR2* plays a pivotal role in regulating human immune responses and inflammatory cascades. Thus, *CYSLTR2* is regarded as a key regulator of immune-inflammatory processes in humans [36]. Preclinical evidence has implicated cysteinyl-leukotriene signaling, including CysLT2R, in intestinal inflammation [37]. A recent cardiovascular study demonstrated that CYSLTR2, acting as an endogenous membrane receptor for C16:0 ceramide, activates Gq protein upon ceramide binding. This activation promotes inflammasome activation and exacerbates atherosclerotic plaque formation [38]. In neurology, studies have indicated that *CYSLTR2* is upregulated in neurodegenerative diseases, such as Alzheimer’s disease, positioning it as a potential therapeutic target [39]. Interestingly, *CYSLTR2* was significantly downregulated in both bioinformatic datasets and clinical PD samples compared with controls in this study, suggesting its potential heterogeneous roles across distinct neurodegenerative diseases. Notably, GSEA revealed that *CYSLTR2* was significantly enriched in the neuroactive ligand–receptor interaction pathway. This pathway encompasses a diverse repertoire of receptors and is critical for physiological processes such as motor control, synaptic transmission, neural excitation–inhibition balance, and learning–memory consolidation. The downregulation of *CYSLTR2* might disrupt neuroactive ligand–receptor crosstalk, potentially contributing to the initiation and progression of PD. Although *CYSLTR2* represents a promising therapeutic target, its clinical translatability requires validation via large-scale clinical trials and mechanistic investigations.

The protein encoded by *TTPAL* (GeneID: 79183) belongs to the tocopherol-transfer protein family. Tocopherol (α), a potent antioxidant, protects cells from oxidative stress-induced damage. Existing studies on *TTPAL* have predominantly focused on oncology. In 2014, *TTPAL* was identified as a candidate oncogene [40], with its overexpression serving as a biomarker for breast cancer [41] and a prognostic indicator for gastric and colorectal cancers [42–44]. GSEA revealed that *TTPAL* was significantly enriched in the pathogenic *E. coli* infection pathway. Intriguingly, patients with PD frequently exhibit intestinal flora dysregulation, with increased relative abundance of pathogenic *E. coli* strains reported in multiple studies [3,45,46]. Such gut-microbiota dysregulation can compromise intestinal-barrier integrity, facilitating the translocation of intestinal pathogens and their metabolites (e.g., lipopolysaccharides) into the bloodstream [47]. This process may subsequently trigger a systemic inflammatory response [48]. Gut dysbiosis, such as pathogenic *E. coli* overgrowth, can activate the intestinal immune system and release pro-inflammatory cytokines (e.g., TNF-α, IL-1β, and IL-6). Consistent with the gut–brain axis hypothesis, such peripheral inflammatory signals can compromise the blood-brain barrier and induce neuroinflammation [49]. Within the central nervous system, pro-inflammatory cytokines activate microglia, induce neuroinflammation, and ultimately contribute to dopaminergic-neuron damage [50]. We hypothesize that *TTPAL* overexpression might disrupt neuronal homeostasis by altering neuronal lipid metabolism, thereby interfering with neurotransmitter synthesis, storage, and release. For instance, dysregulation of lipid homeostasis in dopaminergic neurons impairs dopamine-vesicle packaging and release efficiency, thereby disrupting neural signal transmission and contributing to PD progression. Additionally, this imbalance might compromise neuronal survival and repair mechanisms, rendering neurons more susceptible to damage from oxidative stress and inflammation and accelerating neurodegenerative processes.

By constructing a GGI network, we found that *CYSLTR2* and *TTPAL* interact with members of the GNAQ subfamily (GNA14, GNA15, and GNA11). Previous studies have confirmed that the signaling pathways activated by the GNAQ subfamily can modulate intracellular calcium levels and protein kinase C (PKC) activity [51]. Alterations in calcium-related signaling can further reshape downstream cellular networks [52]; subsequent alterations in calcium levels can alter the function of proteins involved in neurotransmitter release, thereby influencing dopamine release [53]. PKC participates in dopamine-receptor signaling and can regulate dopamine-receptor downregulation and dopamine-transporter internalization; altered PKC signaling has also been implicated in PD [54]. Collectively, these findings suggest that GNAQ-subfamily members can modulate dopamine transporter or receptor function, thereby influencing the dopamine-transport signaling pathway. In recent years, accumulating studies have provided insights into the relationship between the dopamine-signaling pathway and PD. In mouse models, high-frequency deep brain stimulation alleviated parkinsonian motor deficits by desynchronizing GABA release [55]. Additionally, stem-cell-based technology is being explored to replace degenerated dopaminergic neurons within the substantia nigra, with the goal of achieving sustained therapeutic benefits [56]. Overall, these findings provide a basis for elucidating the hierarchical regulatory architecture and feedback mechanisms of the biomarkers, and offer a rationale for targeting the dopamine-receptor signaling pathway to modulate PD progression.

Previous studies have established that PD is characterized by chronic inflammation, with neuroinflammation playing a pivotal role in its pathogenesis. In this study, immune-infiltration analysis revealed six differentially abundant immune-cell types between the PD and control groups, indicating profound alterations in the immune microenvironment in PD. Post-mortem studies in patients with Lewy body disease have revealed increased T-cell infiltration associated with neuroinflammation [57]. Consistently, animal models demonstrate that NKT-cell infiltration drives this inflammatory response, and immunotherapy targeting CD1d can effectively alleviate neurodegeneration [58]. In addition, CD8^+^ T cells play an important role in PD-related pathological processes. First, immunohistochemical analysis of autopsied brain tissues from patients with PD has demonstrated the infiltration of cytotoxic CD8^+^ T cells in the substantia nigra. One neuropathological study suggested that such infiltration may occur prior to detectable α-synuclein aggregation and neuronal death, raising the possibility that CD8^+^ T cells might initiate neuroinflammation at an early pathological stage and contribute to disease onset [59]. Second, single-cell transcriptome and TCR profiling has revealed activated and expanded T-cell populations in PD [60]. In addition, T cells from patients with PD can recognize α-synuclein-derived peptides and mount helper and cytotoxic immune responses [61]. Patients with PD have been reported to exhibit reduced frequencies of memory CD8^+^ T cells, whereas mechanistic experiments suggest that mitophagy contributes to memory CD8 + T-cell formation and survival [62].

This study revealed robust interactions among γδ T cells, NKT cells, and CD8^+^ T cells, suggesting their joint involvement in the progression of PD. Notably, *CYSLTR2* and *TTPAL* expression was correlated with the abundance of CD8^+^ T cells and naïve CD8^+^ T cells, suggesting that these biomarkers might influence PD pathogenesis by modulating the infiltration patterns and compositional balance of specific immune‑cell subsets and subsequently altering the host immune microenvironment. If validated, these associations could inform therapeutic strategies targeting biomarker expression to normalize immune-cell profiles, stabilize neuroinflammation, and ultimately intervene in PD progression.

This study, based on the NetworkAnalyst platform, elucidated the complex regulatory axes governing these biomarkers. E2F1 and TP53 were predicted to jointly regulate the expression of both biomarkers. Studies have found that these TFs play key roles in processes such as cell-cycle regulation, DNA-damage repair, and apoptosis. As a key regulatory factor of the cell cycle, E2F1 not only promotes DNA replication but also plays a crucial role in maintaining genomic stability by facilitating DNA damage repair [63]. In a study of cerebral ischemia–reperfusion injury (CIRI), E2F1-mediated CDK5 activation induced mitochondrial fission while suppressing mitophagy in microglia, thereby triggering inflammation and neuronal apoptosis and exacerbating CIRI severity [64]. E2F1 is known to modulate neuronal apoptosis [65], and existing studies indicate that this pathway plays a critical role in mediating neuronal death specifically in PD models [66]. It is hypothesized that E2F1 promotes PD progression by modulating genes associated with mitochondrial dysfunction, thereby disrupting the respiratory chain and MD. Collectively, these findings provide a basis for achieving controllable therapy by targeting these biomarkers.

Compound screening analysis further underscored the clinical potential of these biomarkers. Among the 36 chemical compounds predicted in this study, 20 exhibited toxic properties, 2 possessed ambiguous functional implications, and 2 represented normal physiological and biochemical compounds endogenous to humans. The remaining 12 compounds exhibited no toxicity or tolerable toxicity profiles and had well-established clinical applications. These agents affect expectoration, diuresis, central nervous system excitation, and immunosuppression, and exert anti-pyretic–analgesic, antioxidant, anti-cancer, and other pharmacological effects. Thus, they represent potential therapeutic agents. Current research indicates that cellulose, catechins, and anti-inflammatory and antioxidant agents exhibit fewer adverse effects. Among these compounds, cellulose effectively targets both biomarkers. Amphetamine can trigger the release of endogenous dopamine in the ventral striatum and medial orbitofrontal cortex. In the context of Parkinson's disease, this dopaminergic response is specifically linked to the development of impulsive-compulsive behavior [31]. However, amphetamine has recognized potential for misuse and may cause neurotoxic effects at high doses, which limits its therapeutic applicability [67].

## 5 Conclusions

By integrating GEO transcriptome data with MDRGs, this study identified two novel biomarkers (*CYSLTR2* and *TTPAL*) associated with MD dysfunction in PD. We systematically characterized these biomarkers, thereby deepening our understanding of PD pathology in the context of MD dysfunction. These findings provide biologically significant targets for dissecting PD pathophysiology and advancing hypothesis‑driven experimental investigations. However, several limitations exist in the present study. First, our machine‑learning models showed limited performance in the independent external cohort, which precludes their use as clinical diagnostic tools. Second, the clinical sample size utilized for RT-qPCR validation was relatively small. Insufficient sample size could reduce statistical power, and the observed expression trends of downregulated CYSLTR2 and upregulated TTPAL might be subject to statistical bias arising from the limited number of samples.

## Supporting information

S1 Fig Diagnostic performance of individual genes and the combined nomogram model. (A) ROC curve analysis in the training set. (B) ROC curve analysis in the validation set. (C) Nomogram. (D) Calibration curve of the nomogram. (E) ROC curve of the nomogram. (F) DCA.
(TIF)

## Abbreviations

DEGs: Differentially expressed genes
GEO: Gene Expression Omnibus
GGI: Gene–gene interaction
GLM: Generalized linear model
GO: Gene Ontology
GSEA: Gene set enrichment analysis
KEGG: Kyoto Encyclopedia of Genes and Genomes
MD: Mitochondrial dynamics
MDRGs: Mitochondrial dynamics-related genes
PD: Parkinson’s disease
PPI: Protein–protein interaction
RF: Random forest
RT-qPCR: Reverse transcription quantitative PCR
SVM: Support vector machine
TFs: Transcription factors

## References

[1] Li M, Ye X, Huang Z, Ye L, Chen C. Global burden of Parkinson’s disease from 1990 to 2021: a population-based study. BMJ Open. 2025;15(4):e095610. doi: 10.1136/bmjopen-2024-095610 40288800 PMC12035419

[2] Ugrumov M. Development of early diagnosis of Parkinson’s disease: Illusion or reality?. CNS Neurosci Ther. 2020;26(10):997–1009. doi: 10.1111/cns.13429 32597012 PMC7539842

[3] Wallen ZD, Demirkan A, Twa G, Cohen G, Dean MN, Standaert DG, et al. Metagenomics of Parkinson’s disease implicates the gut microbiome in multiple disease mechanisms. Nat Commun. 2022;13(1):6958. doi: 10.1038/s41467-022-34667-x 36376318 PMC9663292

[4] Rajan S, Kaas B. Parkinson’s disease: risk factor modification and prevention. Semin Neurol. 2022;42(5):626–38. doi: 10.1055/s-0042-1758780 36427528

[5] Liu T-W, Chen C-M, Chang K-H. Biomarker of Neuroinflammation in Parkinson’s Disease. Int J Mol Sci. 2022;23(8):4148. doi: 10.3390/ijms23084148 35456966 PMC9028544

[6] Zhang X-X, Du J-M, Wang S-Z, Mo X-L, Zhang X-N. Neuronal mitochondrial dynamics: roles in brain disorders and therapeutic potential. Acta Pharmacol Sin. 2026;:10.1038/s41401-025-01746-w. doi: 10.1038/s41401-025-01746-w 41840009

[7] Zhang Y, Li X-W, Zhang Y, Li X. Advances in research on mitochondrial dysfunction in neurodegenerative diseases. J Neurol. 2025;272(5):364. doi: 10.1007/s00415-025-13101-4 40295342

[8] Wang X-L, Feng S-T, Wang Y-T, Yuan Y-H, Li Z-P, Chen N-H, et al. Mitophagy, a form of selective autophagy, plays an essential role in mitochondrial dynamics of parkinson’s disease. Cell Mol Neurobiol. 2022;42(5):1321–39. doi: 10.1007/s10571-021-01039-w 33528716 PMC11421754

[9] Gao X-Y, Yang T, Gu Y, Sun X-H. Mitochondrial dysfunction in parkinson’s disease: from mechanistic insights to therapy. Front Aging Neurosci. 2022;14:885500. doi: 10.3389/fnagi.2022.885500 35795234 PMC9250984

[10] Depierreux F, Parmentier E, Mackels L, Baquero K, Degueldre C, Balteau E, et al. Parkinson’s disease multimodal imaging: F-DOPA PET, neuromelanin-sensitive and quantitative iron-sensitive MRI. NPJ Parkinsons Dis. 2021;7(1):57. doi: 10.1038/s41531-021-00199-2 34238927 PMC8266835

[11] Morbelli S, Garibotto V, Bauckneht M. PET, SPECT, and MRI imaging for evaluation of Parkinson’s disease. Semin Nucl Med. 2024;54(3):151–66. doi: 10.1016/j.semnuclmed.2023.12.004

[12] Waqar Z, Sethi P, Jain D, Singh K, Alsaidan OA, Alzarea SI, et al. Precision medicine in neurodegenerative diseases: genomic approaches to target Amyloid-β, Tau, and Alpha-Synuclein Pathways. Curr Genomics. 2025;26(6):469–94. doi: 10.2174/0113892029372437251010114053 42157911 PMC13154288

[13] Paul S, Tiwari P, Dubey S. Precision enzyme: targeted drug discovery in neurodegenerative disorders. Protein Pept Lett. 2025;32(8):539–56. doi: 10.2174/0109298665391103250825102319 40908696

[14] Khaghani F, Hemmati M, Ebrahimi M, Salmaninejad A. Emerging multi-omic approaches to the molecular diagnosis of mitochondrial disease and available strategies for treatment and prevention. Curr Genomics. 2024;25(5):358–79. doi: 10.2174/0113892029308327240612110334 39323625 PMC11420563

[15] Rath S, Sharma R, Gupta R, Ast T, Chan C, Durham TJ, et al. MitoCarta3.0: an updated mitochondrial proteome now with sub-organelle localization and pathway annotations. Nucleic Acids Res. 2021;49(D1):D1541–7. doi: 10.1093/nar/gkaa1011 33174596 PMC7778944

[16] Smyth GK. Linear models and empirical bayes methods for assessing differential expression in microarray experiments. Stat Appl Genet Mol Biol. 2004;3:Article3. doi: 10.2202/1544-6115.1027 16646809

[17] Wickham H. ggplot2: Elegant Graphics for Data Analysis. 2nd ed. New York: Springer-Verlag. 2016. doi: 10.1007/978-3-319-24277-4

[18] Gu Z, Eils R, Schlesner M. Complex heatmaps reveal patterns and correlations in multidimensional genomic data. Bioinformatics. 2016;32(18):2847–9. doi: 10.1093/bioinformatics/btw313 27207943

[19] Yu G, Wang L-G, Han Y, He Q-Y. clusterProfiler: an R package for comparing biological themes among gene clusters. OMICS. 2012;16(5):284–7. doi: 10.1089/omi.2011.0118 22455463 PMC3339379

[20] Kanehisa M, Furumichi M, Sato Y, Matsuura Y, Ishiguro-Watanabe M. KEGG: biological systems database as a model of the real world. Nucleic Acids Res. 2025;53(D1):D672–7. doi: 10.1093/nar/gkae909 39417505 PMC11701520

[21] Szklarczyk D, Kirsch R, Koutrouli M, Nastou K, Mehryary F, Hachilif R, et al. The STRING database in 2023: protein-protein association networks and functional enrichment analyses for any sequenced genome of interest. Nucleic Acids Res. 2023;51(D1):D638–46. doi: 10.1093/nar/gkac1000 36370105 PMC9825434

[22] Shannon P, Markiel A, Ozier O, Baliga NS, Wang JT, Ramage D, et al. Cytoscape: a software environment for integrated models of biomolecular interaction networks. Genome Res. 2003;13(11):2498–504. doi: 10.1101/gr.1239303 14597658 PMC403769

[23] Kuhn M. Building predictive models in R using the caret package. J Stat Softw. 2008;28(5):1–26. doi: 10.18637/jss.v028.i05

[24] Biecek P. DALEX: explainers for complex predictive models in R. J Mach Learn Res. 2018;19(84):1–5.

[25] Subramanian A, Tamayo P, Mootha VK, Mukherjee S, Ebert BL, Gillette MA, et al. Gene set enrichment analysis: a knowledge-based approach for interpreting genome-wide expression profiles. Proc Natl Acad Sci U S A. 2005;102(43):15545–50. doi: 10.1073/pnas.0506580102 16199517 PMC1239896

[26] Wu T, Hu E, Xu S. clusterProfiler 4.0: a universal enrichment tool for interpreting omics data. Innovation (Camb). 2021;2(3):100141. doi: 10.1016/j.xinn.2021.100141 PMC8454663 34557778

[27] Aran D, Hu Z, Butte AJ. xCell: digitally portraying the tissue cellular heterogeneity landscape. Genome Biol. 2017;18(1):220. doi: 10.1186/s13059-017-1349-1 29141660 PMC5688663

[28] Wei T, Simko V. R package ‘corrplot’: Visualization of a correlation matrix. 2021.

[29] Liu Y, Yang X, Gan J, Chen S, Xiao Z-X, Cao Y. CB-Dock2: improved protein-ligand blind docking by integrating cavity detection, docking and homologous template fitting. Nucleic Acids Res. 2022;50(W1):W159–64. doi: 10.1093/nar/gkac394 35609983 PMC9252749

[30] Livak KJ, Schmittgen TD. Analysis of relative gene expression data using real-time quantitative PCR and the 2(-Delta Delta C(T)) Method. Methods. 2001;25(4):402–8. doi: 10.1006/meth.2001.1262 11846609

[31] Song AK, Hay KR, Trujillo P, Aumann M, Stark AJ, Yan Y, et al. Amphetamine-induced dopamine release and impulsivity in Parkinson’s disease. Brain. 2022;145(10):3488–99. doi: 10.1093/brain/awab487 34951464 PMC10233259

[32] Zhang H, Wang S, Wu P, Hu Z, Chen Y, Guan Y, et al. Revealing the landscape of targeting mitochondrial functions and behaviors to overcome cancer chemoresistance. J Natl Cancer Cent. 2025;5(5):453–73. doi: 10.1016/j.jncc.2025.02.007 41111923 PMC12529618

[33] Shentu J, Xu H, Zhu F. Ammonia-induced lysosomal and mitochondrial damage: a novel perspective on T cell-based cancer immunotherapy. J Natl Cancer Cent. 2025;5(2):105–7. doi: 10.1016/j.jncc.2025.01.004 40265095 PMC12010380

[34] Zhong H, Liu H, Fu Q. Ferroptosis as a therapeutic target in neurodegenerative diseases: exploring the mechanisms and potential of treating alzheimer’s disease and parkinson’s disease. Protein Pept Lett. 2024;31(10):759–72. doi: 10.2174/0109298665333926240927074528 39513303

[35] Barik SK, Bansal R, Singh S. Targeting PI3K/Akt/mTOR with fisetin mechanistic and therapeutics insights for Parkinson’s disease. Letters in Drug Design & Discovery. 2025;22(12):100319. doi: 10.1016/j.lddd.2026.100319

[36] Wang Y, Yang Y, Zhang S, Li C, Zhang L. Modulation of neuroinflammation by cysteinyl leukotriene 1 and 2 receptors: implications for cerebral ischemia and neurodegenerative diseases. Neurobiol Aging. 2020;87:1–10. doi: 10.1016/j.neurobiolaging.2019.12.013 31986345

[37] Sabu Kattuman EE, Teegala LR, Darzi S, Thodeti CK, Paruchuri S. Leukotrienes: bridging the inflammatory gap in asthma and Inflammatory Bowel Diseases (IBD). Compr Physiol. 2025;15(3):e70022. doi: 10.1002/cph4.70022 40568744 PMC12199122

[38] Zhang S, Lin H, Wang J, Rui J, Wang T, Cai Z, et al. Sensing ceramides by CYSLTR2 and P2RY6 to aggravate atherosclerosis. Nature. 2025;641(8062):476–85. doi: 10.1038/s41586-025-08792-8 40049228

[39] Chaudhary A, Singh V, Varadwaj PK, Mani A. Screening natural inhibitors against upregulated G-protein coupled receptors as potential therapeutics of Alzheimer’s disease. J Biomol Struct Dyn. 2022;40(2):673–84. doi: 10.1080/07391102.2020.1817784 32900274

[40] Tuupanen S, Hänninen UA, Kondelin J, von Nandelstadh P, Cajuso T, Gylfe AE, et al. Identification of 33 candidate oncogenes by screening for base-specific mutations. Br J Cancer. 2014;111(8):1657–62. doi: 10.1038/bjc.2014.429 25117815 PMC4200084

[41] Arikan-Soylemez ES, Çilekar M, Akici M, Tokyol Ç, Özyürek HA, Söylemez Z, et al. Investigation of the IL7Rα gene polymorphism rs6897932 and the expression levels of the CDH1, TTPAL, and FHIT genes in patients with breast cancer. P R Health Sci J. 2023;42(4):283–90. 38104284

[42] Liu W, Gou H, Wang X, Li X, Hu X, Su H, et al. TTPAL promotes gastric tumorigenesis by directly targeting NNMT to activate PI3K/AKT signaling. Oncogene. 2021;40(49):6666–79. doi: 10.1038/s41388-021-01838-x 34642500 PMC8660633

[43] Gou H, Liang JQ, Zhang L, Chen H, Zhang Y, Li R, et al. TTPAL promotes colorectal tumorigenesis by stabilizing TRIP6 to activate Wnt/β-Catenin signaling. Cancer Res. 2019;79(13):3332–46. doi: 10.1158/0008-5472.CAN-18-2986 31018940

[44] Arıkan Söylemez ESS, Söylemez Z, Çilekar M, Arıkan Y, Tokyol Ç, Kenger İH, et al. Investigation of the expression levels of CDH1, FHIT, PTEN, and TTPAL genes in colorectal tumors. Turk J Med Sci. 2022;52(1):124–30. doi: 10.3906/sag-2110-296 36161592 PMC10734844

[45] Varesi A, Campagnoli LIM, Fahmideh F, Pierella E, Romeo M, Ricevuti G, et al. The Interplay between Gut Microbiota and Parkinson’s Disease: Implications on Diagnosis and Treatment. Int J Mol Sci. 2022;23(20):12289. doi: 10.3390/ijms232012289 36293176 PMC9603886

[46] Hirayama M, Ohno K. Parkinson’s disease and gut microbiota. Ann Nutr Metab. 2021;77(Suppl. 2):28–35. doi: 10.1159/000518147 34500451

[47] Mohr AE, Crawford M, Jasbi P, Fessler S, Sweazea KL. Lipopolysaccharide and the gut microbiota: considering structural variation. FEBS Lett. 2022;596(7):849–75. doi: 10.1002/1873-3468.14328 35262962

[48] Bhattarai Y, Kashyap PC. Parkinson’s disease: Are gut microbes involved? Am J Physiol Gastrointest Liver Physiol. 2020;319(5):G529–40. doi: 10.1152/ajpgi.00058.2020 32877215 PMC8087343

[49] Chidambaram SB, Essa MM, Rathipriya AG, Bishir M, Ray B, Mahalakshmi AM, et al. Gut dysbiosis, defective autophagy and altered immune responses in neurodegenerative diseases: Tales of a vicious cycle. Pharmacol Ther. 2022;231:107988. doi: 10.1016/j.pharmthera.2021.107988 34536490

[50] Badanjak K, Fixemer S, Smajić S, Skupin A, Grünewald A. The contribution of microglia to neuroinflammation in Parkinson’s Disease. Int J Mol Sci. 2021;22(9):4676. doi: 10.3390/ijms22094676 33925154 PMC8125756

[51] Harden TK, Waldo GL, Hicks SN, Sondek J. Mechanism of activation and inactivation of Gq/phospholipase C-β signaling nodes. Chem Rev. 2011;111(10):6120–9. doi: 10.1021/cr200209p 21988240 PMC3626114

[52] Gorkhali R, Tian L, Dong B, Bagchi P, Deng X, Pawar S, et al. Extracellular calcium alters calcium-sensing receptor network integrating intracellular calcium-signaling and related key pathway. Sci Rep. 2021;11(1):20576. doi: 10.1038/s41598-021-00067-2 34663830 PMC8523568

[53] Yee AG, Forbes B, Cheung P-Y, Martini A, Burrell MH, Freestone PS, et al. Action potential and calcium dependence of tonic somatodendritic dopamine release in the Substantia Nigra pars compacta. J Neurochem. 2019;148(4):462–79. doi: 10.1111/jnc.14587 30203851

[54] Ma H. PKC in the perspective of dopamine receptor signaling. Acta Biochim Pol. 2025;72:14488. doi: 10.3389/abp.2025.14488 40552006 PMC12183092

[55] Xu Z, Duan W, Yuan S, Zhang X, You C, Yu J-T, et al. Deep brain stimulation alleviates Parkinsonian motor deficits through desynchronizing GABA release in mice. Nat Commun. 2025;16(1):3726. doi: 10.1038/s41467-025-59113-6 40253429 PMC12009282

[56] Tabar V, Sarva H, Lozano AM. Phase I trial of hES cell-derived dopaminergic neurons for Parkinson's disease. Nature. 2025;641(8064):978–83. doi: 10.1038/s41586-025-08845-y PMC12095069 40240592

[57] Iba M, Kim C, Sallin M, Kwon S, Verma A, Overk C, et al. Neuroinflammation is associated with infiltration of T cells in Lewy body disease and α-synuclein transgenic models. J Neuroinflammation. 2020;17(1):214. doi: 10.1186/s12974-020-01888-0 32680537 PMC7368752

[58] Iba M, Kwon S, Kim C, Szabo M, Horan-Portelance L, Lopez-Ocasio M, et al. Immunotherapy with an antibody against CD1d modulates neuroinflammation in an α-synuclein transgenic model of Lewy body like disease. J Neuroinflammation. 2024;21(1):93. doi: 10.1186/s12974-024-03087-7 38622654 PMC11017481

[59] Galiano-Landeira J, Torra A, Vila M, Bové J. CD8 T cell nigral infiltration precedes synucleinopathy in early stages of Parkinson’s disease. Brain. 2020;143(12):3717–33. doi: 10.1093/brain/awaa269 33118032

[60] Wang P, Yao L, Luo M, Zhou W, Jin X, Xu Z, et al. Single-cell transcriptome and TCR profiling reveal activated and expanded T cell populations in Parkinson’s disease. Cell Discov. 2021;7(1):52. doi: 10.1038/s41421-021-00280-3 34282123 PMC8289849

[61] Sulzer D, Alcalay RN, Garretti F, Cote L, Kanter E, Agin-Liebes J, et al. T cells from patients with Parkinson’s disease recognize α-synuclein peptides. Nature. 2017;546(7660):656–61. doi: 10.1038/nature22815 28636593 PMC5626019

[62] Franco F, Bevilacqua A, Wu R-M, Kao K-C, Lin C-P, Rousseau L, et al. Regulatory circuits of mitophagy restrict distinct modes of cell death during memory CD8+ T cell formation. Sci Immunol. 2023;8(87):eadf7579. doi: 10.1126/sciimmunol.adf7579 37738363

[63] Choi E-H, Kim KP. E2F1 facilitates DNA break repair by localizing to break sites and enhancing the expression of homologous recombination factors. Exp Mol Med. 2019;51(9):1–12. doi: 10.1038/s12276-019-0307-2 31534120 PMC6802646

[64] Yuan Y-J, Chen T, Yang Y-L, Han H-N, Xu L-M. E2F1/CDK5/DRP1 axis mediates microglial mitochondrial division and autophagy in the pathogenesis of cerebral ischemia-reperfusion injury. Clin Transl Med. 2025;15(2):e70197. doi: 10.1002/ctm2.70197 39968698 PMC11836619

[65] Hou ST, Callaghan D, Fournier MC, Hill I, Kang L, Massie B, et al. The transcription factor E2F1 modulates apoptosis of neurons. J Neurochem. 2000;75(1):91–100. doi: 10.1046/j.1471-4159.2000.0750091.x 10854251

[66] Das AK, Biswas SC. Cdc25A phosphatase is activated and mediates neuronal cell death by PUMA via pRb/E2F1 pathway in a model of Parkinson’s disease. Biochim Biophys Acta Mol Cell Res. 2024;1871(8):119848. doi: 10.1016/j.bbamcr.2024.119848 39305937

[67] Berman SM, Kuczenski R, McCracken JT, London ED. Potential adverse effects of amphetamine treatment on brain and behavior: a review. Mol Psychiatry. 2009;14(2):123–42. doi: 10.1038/mp.2008.90 18698321 PMC2670101

