# Supplementary material for: Identification and validation of biomarkers associated with mitochondrial dynamics dysfunction in Parkinson’s disease based on GEO datasets
Source: PLoS One. 2026 Nov 4;21(11):e0362081. doi: 10.1371/journal.pone.0362081 (PMC13734593; doi:10.1371/journal.pone.0362081)
Supplement: Peer review history

# Peer review: Decision Letter 0

**DOI:** 10.1371/journal.pone.0362081.r001

**Authors:** Zhijie Xu

11 Jun 2026

Dear Dr. Chen,

Thank you for submitting your manuscript to PLOS ONE. After careful consideration, we feel that it has merit but does not fully meet PLOS ONE’s publication criteria as it currently stands. Therefore, we invite you to submit a revised version of the manuscript that addresses the points raised during the review process.

Please submit your revised manuscript by Aug 10 2026 11:59PM. If you will need more time than this to complete your revisions, please reply to this message or contact the journal office at plosone@plos.org. When you're ready to submit your revision, log on to https://www.editorialmanager.com/pone/ and select the 'Submissions Needing Revision' folder to locate your manuscript file.

- A letter that responds to each point raised by the academic editor and reviewer(s). You should upload this letter as a separate file labeled 'Response to Reviewers'.
- A marked-up copy of your manuscript that highlights changes made to the original version. You should upload this as a separate file labeled 'Revised Manuscript with Track Changes'.
- An unmarked version of your revised paper without tracked changes. You should upload this as a separate file labeled 'Manuscript'.

If you would like to make changes to your financial disclosure, please include your updated statement in your cover letter. Guidelines for resubmitting your figure files are available below the reviewer comments at the end of this letter.

If applicable, we recommend that you deposit your laboratory protocols in protocols.io to enhance the reproducibility of your results. Protocols.io assigns your protocol its own identifier (DOI) so that it can be cited independently in the future. For instructions see: https://journals.plos.org/plosone/s/submission-guidelines#loc-laboratory-protocols. Additionally, PLOS ONE offers an option for publishing peer-reviewed Lab Protocol articles, which describe protocols hosted on protocols.io. Read more information on sharing protocols at https://plos.org/protocols?utm_medium=editorial-email&utm_source=authorletters&utm_campaign=protocols.

As the corresponding author, your ORCID iD is verified in the submission system and will appear in the published article. PLOS supports the use of ORCID, and we encourage all coauthors to register for an ORCID iD and use it as well. Please encourage your coauthors to verify their ORCID iD within the submission system before final acceptance, as unverified ORCID iDs will not appear in the published article. *Only*  the individual author can complete the verification step; PLOS staff *cannot*  verify ORCID iDs on behalf of authors.

We look forward to receiving your revised manuscript.

Kind regards,

Zhijie Xu

Academic Editor

PLOS One

**Journal Requirements:**

1. When submitting your revision, we need you to address these additional requirements.

Please ensure that your manuscript meets PLOS ONE's style requirements, including those for file naming. The PLOS ONE style templates can be found at

https://journals.plos.org/plosone/s/file?id=wjVg/PLOSOne_formatting_sample_main_body.pdf and

https://journals.plos.org/plosone/s/file?id=ba62/PLOSOne_formatting_sample_title_authors_affiliations.pdf

2. Thank you for stating the following in the Acknowledgments Section of your manuscript:

We would like to express our sincere gratitude to all individuals and organizations who supported and assisted us throughout this research. Special thanks to my wife: Shimian Liu. In conclusion, we extend our thanks to everyone who has supported and assisted us along the way. Without your support, this research would not have been possible.

We note that you have provided funding information that is not currently declared in your Funding Statement. However, funding information should not appear in the Acknowledgments section or other areas of your manuscript. We will only publish funding information present in the Funding Statement section of the online submission form.

Please remove any funding-related text from the manuscript and let us know how you would like to update your Funding Statement. Currently, your Funding Statement reads as follows:

The author(s) received no specific funding for this work.

Please include your amended statements within your cover letter; we will change the online submission form on your behalf.

3. PLOS requires an ORCID iD for the corresponding author in Editorial Manager on papers submitted after December 6th, 2016. Please ensure that you have an ORCID iD and that it is validated in Editorial Manager. To do this, go to ‘Update my Information’ (in the upper left-hand corner of the main menu), and click on the Fetch/Validate link next to the ORCID field. This will take you to the ORCID site and allow you to create a new iD or authenticate a pre-existing iD in Editorial Manager.

4. Please upload a new copy of Figures 1, 3, 4, 5 and 6, as the detail is not clear. Please follow the link for more information:  https://journals.plos.org/plosone/s/figures

5. If the reviewer comments include a recommendation to cite specific previously published works, please review and evaluate these publications to determine whether they are relevant and should be cited. There is no requirement to cite these works unless the editor has indicated otherwise.

[Note: HTML markup is below. Please do not edit.]

Reviewers' comments:

**Comments to the Author**

1. Is the manuscript technically sound, and do the data support the conclusions?

Reviewer #1: Partly

Reviewer #2: Yes

**********

2. Has the statistical analysis been performed appropriately and rigorously?

Reviewer #1: Yes

Reviewer #2: Yes

**********

3. Have the authors made all data underlying the findings in their manuscript fully available?

The PLOS Data policy

Reviewer #1: Yes

Reviewer #2: Yes

**********

4. Is the manuscript presented in an intelligible fashion and written in standard English?

Reviewer #1: Yes

Reviewer #2: Yes

**********

**Reviewer #1:**  1. No cross-validation strategy or independent test split is described. ROC curves with AUC values for each model (RF, SVM, GLM) in both training and validation sets should be reported to quantify diagnostic performance.

2. n=5 per group is insufficient to support the reported significance (P<0.0001). Authors should expand clinical validation to at least 20–30 subjects per group.

3. The use of Spearman-ranked gene lists as GSEA input should be explicitly described as a preranked approach. Permutation number, FDR threshold, and leading-edge genes should be reported.

4. A Vina score of −5.9 kcal/mol is relatively weak. Authors should benchmark this against known CYSLTR2 endogenous ligands and present key interacting residues in tabular form to support the biological relevance of the docking result.

5. Duplicate period at the end of Section 2.12.

6. "Escherichia coli" should be italicized and written in lowercase throughout.

7. Figure 4 panel descriptions in the legend do not correspond to the order described in the Results text.

**Reviewer #2:**  This manuscript addresses an interesting and potentially useful topic by identifying candidate biomarkers for Parkinson’s disease using public transcriptomic datasets and machine-learning approaches. The overall workflow is reasonable, and the additional RT-qPCR validation adds some value. However, several methodological and interpretative issues should be addressed.

1, the relationship between the identified biomarkers and mitochondrial dynamics should be described more cautiously. CYSLTR2 and TTPAL were identified through a mitochondrial dynamics-related scoring and DEG screening framework, rather than being directly demonstrated as core regulators of mitochondrial fission, fusion, mitophagy, mitochondrial transport, or mitochondrial quality control. Therefore, I suggest revising the wording throughout the manuscript. For example, terms such as “mitochondrial dynamics biomarkers” could be replaced with “mitochondrial dynamics-associated candidate biomarkers” or “candidate biomarkers identified through a mitochondrial dynamics-related transcriptomic framework.”

2, the machine-learning procedure should be described in greater detail. The authors should clarify the data preprocessing steps, feature-selection process, cross-validation strategy, hyperparameter tuning, and how overfitting was minimized, especially given the relatively small size of the training cohort.

3, the diagnostic performance of the final biomarkers should be more clearly presented. ROC curves, AUC values with confidence intervals, sensitivity, specificity, and preferably validation-set performance should be reported for CYSLTR2, TTPAL, and the combined model.

4, the RT-qPCR validation cohort is very small, including only 5 PD patients and 5 controls. The authors should acknowledge this as a preliminary validation and avoid overinterpreting the clinical applicability of the biomarkers. If possible, additional clinical samples would strengthen the study.

5, the manuscript would benefit from language polishing and correction of minor typographical and formatting issues.

**********

what does this mean?). If published, this will include your full peer review and any attached files.

If you choose “no”, your identity will remain anonymous but your review may still be made public.

**Do you want your identity to be public for this peer review?**  For information about this choice, including consent withdrawal, please see our Privacy Policy

Reviewer #1: No

Reviewer #2: No

**********

[NOTE: If reviewer comments were submitted as an attachment file, they will be attached to this email and accessible via the submission site. Please log into your account, locate the manuscript record, and check for the action link "View Attachments". If this link does not appear, there are no attachment files.]

To ensure your figures meet our technical requirements, please review our figure guidelines: https://journals.plos.org/plosone/s/figures

You may also use PLOS’s free figure tool, NAAS, to help you prepare publication quality figures: https://journals.plos.org/plosone/s/figures#loc-tools-for-figure-preparation.

NAAS will assess whether your figures meet our technical requirements by comparing each figure against our figure specifications.

# Author comment: Author response to Decision Letter 1

**DOI:** 10.1371/journal.pone.0362081.r002

25 Aug 2026

Point-by-point response to the reviewer comments

Dear Editor and Reviewers,

We appreciate your valuable comments and suggestions, which have greatly improved this manuscript. The manuscript has been carefully revised in accordance with all your requirements, and detailed point-by-point responses are provided below. We hope the revised version meets the journal’s publication criteria. Please do not hesitate to contact us if you require further information.

Journal Requirements:

1. When submitting your revision, we need you to address these additional requirements.

Please ensure that your manuscript meets PLOS ONE's style requirements, including those for file naming. The PLOS ONE style templates can be found at

https://journals.plos.org/plosone/s/file?id=wjVg/PLOSOne_formatting_sample_main_body.pdf and https://journals.plos.org/plosone/s/file?id=ba62/PLOSOne_formatting_sample_title_authors_affiliations.pdf

Reply:

We have carefully reviewed the PLOS ONE formatting guidelines and the two official template documents. The entire manuscript has been adjusted in accordance with these requirements. We standardized heading styles, author affiliations, corresponding author labelling, figure citations, reference formatting and supplementary material annotations. In addition, all uploaded files are named following the journal’s specifications to fully comply with PLOS ONE submission standards. Thank you for the reminder.

2. Thank you for stating the following in the Acknowledgments Section of your manuscript. We would like to express our sincere gratitude to all individuals and organizations who supported and assisted us throughout this research. Special thanks to my wife: Shimian Liu. In conclusion, we extend our thanks to everyone who has supported and assisted us along the way. Without your support, this research would not have been possible.

We note that you have provided funding information that is not currently declared in your Funding Statement. However, funding information should not appear in the Acknowledgments section or other areas of your manuscript. We will only publish funding information present in the Funding Statement section of the online submission form.

Please remove any funding-related text from the manuscript and let us know how you would like to update your Funding Statement. Currently, your Funding Statement reads as follows: The author(s) received no specific funding for this work.

Please include your amended statements within your cover letter; we will change the online submission form on your behalf.

Reply:

We appreciate the editorial reminder regarding the Acknowledgments and Funding Statement. Corresponding revisions have been made as required:

The Acknowledgments section has been revised (Page 29–30, Lines 671–676). The updated text only acknowledges researchers of public databases, clinical participants and family members. No funding-related content appears anywhere in the manuscript.

No specific funding supported this work. The existing funding statement, The author(s) received no specific funding for this work, remains unchanged, so no updates to the Funding Statement in the submission system are needed.

These revisions comply with PLOS ONE requirements separating acknowledgments from formal funding declarations. Thank you for your careful guidance.

3. PLOS requires an ORCID ID for the corresponding author in Editorial Manager on papers submitted after December 6th, 2016. Please ensure that you have an ORCID ID and that it is validated in Editorial Manager. To do this, go to ‘Update my Information’ (in the upper left-hand corner of the main menu), and click on the Fetch/Validate link next to the ORCID field. This will take you to the ORCID site and allow you to create a new ID or authenticate a pre-existing ID in Editorial Manager.

Reply:

We appreciate the editorial reminder regarding the ORCID requirement for the corresponding author. The corresponding author, Jinjin Chen, holds a valid ORCID identifier (0009-0007-5960-5652). We will log into Editorial Manager, navigate to the "Update my Information" page, and click the Fetch/Validate link beside the ORCID field to complete authentication, ensuring the ORCID ID is successfully validated within the submission system. This operation fulfills the mandatory ORCID requirement specified by PLOS ONE. Thank you for your careful guidance.

4. Please upload a new copy of Figures 1, 3, 4, 5 and 6, as the detail is not clear. Please follow the link for more information: https://journals.plos.org/plosone/s/figures.

Reply:

We appreciate the editorial reminder that Figures 1, 3, 4, 5 and 6 lack sufficient clarity. These figures have been reworked in strict accordance with PLOS ONE’s figure guidelines. Image resolution has been optimized to meet the journal’s requirements (≥300 dpi), and revised high-quality figure files will be uploaded together with the revised manuscript. Thank you for your careful guidance.

5. If the reviewer comments include a recommendation to cite specific previously published works, please review and evaluate these publications to determine whether they are relevant and should be cited. There is no requirement to cite these works unless the editor has indicated otherwise.

Reply:

We appreciate this editorial reminder. We have carefully reviewed the journal’s guidance regarding suggested citations. All publications recommended by the reviewers have been assessed for relevance to our work. Highly relevant articles have been cited in the revised manuscript. References with limited relevance will not be included, with rationales provided in the response letter where applicable. We adhere to journal citation standards to ensure all cited literature is pertinent. Thank you for your guidance.

Reviewers’s comments

Reviewer #1:

Comment 1. No cross-validation strategy or independent test split is described. ROC curves with AUC values for each model (RF, SVM, GLM) in both training and validation sets should be reported to quantify diagnostic performance.

Reply:

Thank you for this important comment. We agree that the description of validation strategy and diagnostic performance of machine‑learning models was insufficient in the original manuscript.

Regarding the model validation strategy, it should be noted that GSE6613 (n = 72) was used as the model‑building cohort, and the fully independent dataset GSE99039 (n = 438) served as the external validation cohort. Dataset partitioning has been explicitly clarified in the revised manuscript (Page 6, Lines 146‑148). Given the relatively limited sample size of GSE6613, further splitting it into a training subset and an internal test subset would substantially reduce the sample size available for both model training and testing, which may increase the instability of model performance estimation. Therefore, we did not create a fixed‑proportion hold‑out test subset within GSE6613. Instead, 5‑fold stratified cross‑validation was performed in this cohort for internal evaluation, while GSE99039 was used as an independent external test cohort. This design balances full utilization of training data and independent assessment of model generalizability. Specifically, disease status (PD versus healthy control) was defined as the binary outcome, and expression levels of candidate biomarkers were used as input features. Random forest (RF), support vector machine (SVM), and generalized linear model (GLM) were constructed separately. Five‑fold stratified cross‑validation maintains roughly balanced proportions of PD and healthy control samples within each fold, thereby reducing bias caused by a single random data split. Descriptions of this cross‑validation strategy have been added to the Methods section of the revised manuscript (Page7-8, Lines149 -195).

Meanwhile, ROC curves and corresponding AUC values for the three machine‑learning models have been supplemented. The AUC values obtained by direct fitting on the training cohort were 1.000 (95% CI: 1.000–1.000) for random forest, 0.997 (95% CI: 0.991–1.000) for support vector machine, and 0.990 (95% CI: 0.975–1.000) for generalized linear model. Considering the potential optimistic bias from direct fitting on the training set, we further calculated AUC values from 5‑fold stratified cross‑validation: 0.839 (95% CI: 0.786–0.891) for random forest, 0.818 (95% CI: 0.713–0.923) for generalized linear model, and 0.925 (95% CI: 0.887–0.962) for support vector machine (Page15-16, Lines 347-366).

Subsequently, the above‑mentioned models were applied to the independent external cohort GSE99039, yielding AUC values of approximately 0.55‑0.59, which were markedly lower than those from the training cohort and internal cross‑validation. These results indicate limited discriminatory power and generalizability of the models in an independent cohort, suggesting that they cannot yet serve as robust diagnostic tools for PD. Technical platforms, sample composition, clinical characteristics and disease heterogeneity between the training and external cohorts may collectively contribute to the cross‑cohort performance of the models (Page16, Lines 366-368).

It is noteworthy that the primary purpose of machine‑learning models in this study was to assist in screening candidate genes such as CYSLTR2 and TTPAL, rather than developing predictive tools ready for direct clinical diagnosis. External validation results suggest that these candidate genes and their combinations require further validation in larger independent cohorts with consistent technical platforms and complete clinical information.

Comment 2. n=5 per group is insufficient to support the reported significance (P<0.0001). Authors should expand clinical validation to at least 20–30 subjects per group.

Reply:

We appreciate this professional comment. We agree that the clinical cohort with only five subjects per group limits the generalizability of our RT-qPCR findings, and we recognize the merit of expanding the cohort to 20–30 participants per group as suggested. However, due to objective constraints including limited recruitment time, scarcity of eligible patients and sample access requirements, we cannot enlarge the clinical validation cohort within the revision period.

Corresponding revisions have been implemented: We have moderated the interpretation of RT-qPCR expression differences within the Abstract and Results to avoid overinterpretation of our findings. In addition, we have substantially expanded the Limitations section to discuss insufficient statistical power and potential bias caused by the small sample size, and explicitly state that these RT-qPCR results constitute preliminary findings requiring further verification in larger independent cohorts (Page 21, Lines 477–483; Page 27–28, Lines 642–646). These revisions clarify the scope of our conclusions and enhance the rigor of data interpretation. Thank you for your careful guidance.

Comment 3. The use of Spearman-ranked gene lists as GSEA input should be explicitly described as a preranked approach. Permutation number, FDR threshold, and leading-edge genes should be reported.

Reply：

We appreciate this valuable comment. Corresponding supplements have been made to the Methods section. We adopted a preranked GSEA approach. For each biomarker, Spearman correlation coefficients with genome-wide genes were calculated and used as the ranking metric. GSEA was implemented via the R clusterProfiler package against the KEGG pathway gene set. A total of 10,000 permutations were performed, and multiple testing was corrected using the Benjamini–Hochberg method. Pathways with FDR < 0.05 and |NES| > 1 were defined as significantly enriched, and leading-edge genes from enriched pathways were extracted for subsequent analysis (Pages 9, Lines 210–219). These revisions clarify the analytical framework and enhance the reproducibility of our bioinformatic workflow. Thank you for your careful guidance.

Comment 4. A Vina score of −5.9 kcal/mol is relatively weak. Authors should benchmark this against known CYSLTR2 endogenous ligands and present key interacting residues in tabular form to support the biological relevance of the docking result.

Reply:

Thank you for this valuable comment. We agree that the original Vina score alone cannot sufficiently support the biological relevance of the predicted interaction between amphetamine and CYSLTR2. We have therefore performed additional comparative analyses against endogenous ligands as suggested.

In the revised work, well‑characterized endogenous ligands of CYSLTR2, including leukotriene C4 (LTC4), leukotriene D4 (LTD4), and leukotriene E4 (LTE4), were docked onto CYSLTR2 using the CB‑Dock2 platform under identical computational conditions for direct comparison with amphetamine. The updated docking yielded a predicted binding affinity of −6.6 kcal/mol for amphetamine. The corresponding Vina scores for LTC4, LTD4, and LTE4 were −8.9 kcal/mol, −7.7 kcal/mol, and −7.8 kcal/mol, respectively. These results indicate that amphetamine exhibits weaker predicted binding affinity compared with these endogenous ligands.

Further binding‑mode analysis revealed that amphetamine accesses the predicted binding pocket of CYSLTR2 and forms an interaction with residue SER169 at a distance of 2.6 Å. Key interacting residues and interaction distances for the three endogenous ligands were also systematically characterized. Predicted binding affinities, critical interacting residues, and interaction distances for amphetamine, LTC4, LTD4, and LTE4 have been summarized in Table 2 (“Predicted binding affinities and key interacting residues of amphetamine and endogenous ligands with CYSLTR2”) in the revised manuscript (Pages 18–19, Lines 435–448).

Based on these findings, we have moderated our statements concerning amphetamine‑CYSLTR2 binding. We no longer claim strong binding affinity, and instead only suggest a potential structural interaction. We also emphasize that these observations are purely in silico docking predictions, and their biological relevance requires further validation via in‑vitro binding and functional assays. This supplementary benchmarking strengthens the interpretive rigor of our molecular docking results.

Comment 5. Duplicate period at the end of Section 2.12.

Reply:

We appreciate your careful review. We have checked Section 2.12(Pages 12–13, Lines 288–292) and removed the redundant duplicate period at the end of this section. We have also inspected the full text of this subsection to avoid similar punctuation errors. This revision standardizes textual formatting. Thank you for your careful guidance.

Comment 6. "Escherichia coli" should be italicized and written in lowercase throughout.

Reply:

We appreciate this careful comment. We have systematically searched all instances of Escherichia coli across the full manuscript and revised them in accordance with taxonomic conventions. The genus name starts with an uppercase letter, the species epithet is lowercase, and the binomial name is consistently italicized throughout the text. This revision standardizes the formatting of Latin species names and improves the academic rigor of the manuscript. Thank you for your careful guidance.

Comment 7. Figure 4 panel descriptions in the legend do not correspond to the order described in the Results text.

Reply:

We appreciate this valuable comment. We have identified the mismatch between the Figure 4 legend and the description in the Results section. Originally, GSEA and immune infiltration analyses were presented in separate subsections, which conflicted with the panel order of Figure 4. We have combined the original Sections 3.4 and 3.5 and restructured the text following the panel sequence a→b→c→d→e→f of Figure 4. The description in the Results now matches the panel order in the figure legend (Pages 17-18, Lines 390–415). This revision aligns textual description with figure presentation and improves the logical flow of the Results. Thank you for your careful guidance.

Reviewer #2: This manuscript addresses an interesting and potentially useful topic by identifying candidate biomarkers for Parkinson’s disease using public transcriptomic datasets and machine-learning approaches. The overall workflow is reasonable, and the additional RT-qPCR validation adds some value. However, several methodological and interpretative issues should be addressed.

Comment 1. the relationship between the identified biomarkers and mitochondrial dynamics should be described more cautiously. CYSLTR2 and TTPAL were identified through a mitochondrial dynamics-related scoring and DEG screening framework, rather than being directly demonstrated as core regulators of mitochondrial fission, fusion, mitophagy, mitochondrial transport, or mitochondrial quality control. Therefore, I suggest revising the wording throughout the manuscript. For example, terms such as “mitochondrial dynamics biomarkers” could be replaced with “mitochondrial dynamics-associated candidate biomarkers” or “candidate biomarkers identified through a mitochondrial dynamics-related transcriptomic framework.”

Reply:

We appreciate this professional and detailed comment. We fully recognize that the original descriptions regarding the association between CYSLTR2/TTPAL and mitochondrial dynamics lacked sufficient rigor, and systematic revisions have been implemented throughout the manuscript.

CYSLTR2 and TTPAL are MDRG-correlated DEGs rather than canonical mitochondrial dynamics regulators (MDRGs). They were screened via a transcriptomic framework based on GSVA scores of mitochondrial dynamics-related genes. To date, direct experimental evidence confirming them as core regulators of mitochondrial fission, fusion, mitophagy, mitochondrial transport or mitochondrial quality control is lacking. Following your suggestion, we removed the ambiguous phrase “mitochondrial dynamics biomarkers” throughout the manuscript. Inspired by your recommended term mitochondrial dynamics-associated candidate biomarkers, we adopted the semantically compatible expression biomarkers associated with mitochondrial dynamics dysfunction. This wording clarifies that these markers are linked to pathological mitochondrial abnormalities, instead of being core mitochondrial dynamics regulators. The manuscript title has been adjusted accordingly to clarify this conceptual distinction:

Original title: Identification and validation of biomarkers associated with mitochondrial dynamics in Parkinson's disease based on the GEO dataset

Revised title: Identification and validation of biomarkers associated with mitochondrial dynamics dysfunction in Parkinson's disease based on GEO datasets

These revisions eliminate conceptual confusion, strengthen logical reasoning and improve the rigor of academic statements, preventing potential misunderstanding among readers. Thank you for your careful guidance.

Comment 2. the machine-learning procedure should be described in greater detail. The authors should clarify the data preprocessing steps, feature-selection process, cross-validation strategy, hyperparameter tuning, and how overfitting was minimized, especially given the relatively small size of the training cohort.

Reply:

We appreciate this valuable comment. We have supplemented detailed descriptions of the machine-learning workflow, including data preprocessing, feature selection, cross-validation strategy, hyperparameter configuration and overfitting mitigation. Considering the limited sample size of the training cohort, relevant methodological quality-control details have been added to the Methods section (Pages 6–8, Lines 143–195).

Before modeling, missing value imputation, batch correction and Z-score normalization were performed on the gene expression matrix. The 17 candidate genes were included as features, and disease status served as the binary outcome variable. Random forest, support vector machine and generalized linear model were constructed using the R caret package. Stratified 5-fold cross-validation was adopted to balance positive and negative samples in each fold and reduce bias caused by random partitioning. Feature subsets were preliminarily screened according to variable importance metrics of each model. Residual diagnostics were performed for the GLM, and the optimal model was selected based on cross-validation performance across all algorithms, which was further validated by expression trends in training and validation cohorts. Models were trained with default hyperparameters implemented in the package, and stratified cross-validation was used to evaluate model stability. Meanwhile, stratified cross-validation, moderate model complexity restriction and cross-comparison among multiple algorithms were jointly used to mitigate potential overfitting risks arising from the relatively small training dataset. These revisions improve the transparency and reproducibility of our machine-learning pipeline and strengthen the reliability of our findings. Thank you for your careful guidance.

Comment 3. the diagnostic performance of the final biomarkers should be more clearly presented. ROC curves, AUC values with confidence intervals, sensitivity, specificity, and preferably validation-set performance should be reported for CYSLTR2, TTPAL, and the combined model.

Reply:

We appreciate this valuable comment regarding the presentation of diagnostic performance. We have evaluated CYSLTR2, TTPAL, and their combined model in both training and validation sets. ROC curves were generated, and AUC values with 95% confidence intervals, sensitivity and specificity were calculated and reported for each marker. The AUCs of individual genes ranged from 0.549 to 0.667 (all below 0.7), indicating limited discriminatory ability when used alone. We constructed a combined nomogram incorporating CYSLTR2 and TTPAL, which was also validated in both cohorts and achieved an AUC of 0.707. Calibration curves demonstrated favorable consistency between predicted and observed outcomes. Decision curve analysis (DCA) further revealed that the combined nomogram yielded higher clinical net benefit compared with single-gene markers (Page 16, Lines 379–389, Supplementary Figure 1). All diagnostic metrics have been systematically organized for clearer visualization. Thank you for your careful guidance.

Comment 4. the RT-qPCR validation cohort is very small, including only 5 PD patients and 5 controls. The authors should acknowledge this as a preliminary validation and avoid overinterpreting the clinical applicability of the biomarkers. If possible, additional clinical samples would strengthen the study.

Reply:

We appreciate this professional comment. We agree that the RT-qPCR validation cohort consisting of only five PD patients and five controls has a limited sample size. However, due to objective constraints including limited recruitment time, scarcity of eligible patients and sample access requirements, we cannot recruit additional clinical samples during this revision.

We have moderated relevant interpretations throughout the manuscript: descriptions regarding the clinical utility of biomarkers have been tempered in the Abstract and Results to avoid overinterpretation. Furthermore, we have expanded the Limitations section to explicitly state that these RT-qPCR findings represent preliminary validation, and further verification in larger independent clinical cohorts is required (Page 27–28, Lines 642–646). These revisions define the appropriate scope of our conclusions and improve the rigor of data interpretation. Thank you for your careful guidance.

Comment 5. the manuscript would benefit from language polishing and correction of minor typographical and formatting issues.

Reply：

We appreciate this valuable comment. Comprehensive revisions regarding language, typography and formatting have been completed. We have conducted a full self-proofread to identify and correct errors in spelling, punctuation, layout and grammar throughout the manuscript. In addition, systematic language polishing has been performed by a professional English-editing service to standardize academic expressions, streamline sentence logic and optimize word choice. A language editing certificate is available upon request. These revisions improve the readability and formatting consistency of the manuscript. Thank you for your careful feedback.

Attachment Submitted filename: Response to Reviewers.docx

# Peer review: Decision Letter 1

**DOI:** 10.1371/journal.pone.0362081.r003

**Authors:** Ramon Trullas

20 Oct 2026

Identification and validation of biomarkers associated with mitochondrial dynamics dysfunction in Parkinson’s disease based on GEO datasets

PONE-D-26-24194R1

Dear Dr. Chen,

We’re pleased to inform you that your manuscript has been judged scientifically suitable for publication and will be formally accepted for publication once it meets all outstanding technical requirements.

Within one week, you’ll receive an e-mail detailing the required amendments. When these have been addressed, you’ll receive a formal acceptance letter and your manuscript will be scheduled for publication.

An invoice will be generated when your article is formally accepted. Please note, if your institution has a publishing partnership with PLOS and your article meets the relevant criteria, all or part of your publication costs will be covered. Please make sure your user information is up-to-date by logging into Editorial Manager at Editorial Manager® and clicking the ‘Update My Information' link at the top of the page. For questions related to billing, please contact billing support.

If your institution or institutions have a press office, please notify them about your upcoming paper to help maximize its impact. If they’ll be preparing press materials, please inform our press team as soon as possible -- no later than 48 hours after receiving the formal acceptance. Your manuscript will remain under strict press embargo until 2 pm Eastern Time on the date of publication. For more information, please contact onepress@plos.org.

Kind regards,

Ramon Trullas

Academic Editor

PLOS One

Additional Editor Comments (optional):

All comments have been addressed

Reviewers' comments:

Reviewer's Responses to Questions

**Comments to the Author**

Reviewer #1: All comments have been addressed

**********

2. Is the manuscript technically sound, and do the data support the conclusions?

Reviewer #1: Yes

**********

3. Has the statistical analysis been performed appropriately and rigorously?

Reviewer #1: Yes

**********

4. Have the authors made all data underlying the findings in their manuscript fully available?

The PLOS Data policy

Reviewer #1: Yes

**********

5. Is the manuscript presented in an intelligible fashion and written in standard English?

Reviewer #1: Yes

**********

Reviewer #1: (No Response)

**********

what does this mean?). If published, this will include your full peer review and any attached files.

If you choose “no”, your identity will remain anonymous but your review may still be made public.

**Do you want your identity to be public for this peer review?**  For information about this choice, including consent withdrawal, please see our Privacy Policy

Reviewer #1: No

**********

# Editor report: Acceptance letter

**DOI:** 10.1371/journal.pone.0362081.r004

**Authors:** Ramon Trullas

PONE-D-26-24194R1

PLOS One

Dear Dr. Chen,

I'm pleased to inform you that your manuscript has been deemed suitable for publication in PLOS One. Congratulations! Your manuscript is now being handed over to our production team.

At this stage, our production department will prepare your paper for publication. This includes ensuring the following:

* All references, tables, and figures are properly cited

* All relevant supporting information is included in the manuscript submission,

* There are no issues that prevent the paper from being properly typeset

You will receive further instructions from the production team, including instructions on how to review your proof when it is ready. Please keep in mind that we are working through a large volume of accepted articles, so please give us a few days to review your paper and let you know the next and final steps.

Lastly, if your institution or institutions have a press office, please let them know about your upcoming paper now to help maximize its impact. If they'll be preparing press materials, please inform our press team within the next 48 hours. Your manuscript will remain under strict press embargo until 2 pm Eastern Time on the date of publication. For more information, please contact onepress@plos.org.

You will receive an invoice from PLOS for your publication fee after your manuscript has reached the completed accept phase. If you receive an email requesting payment before acceptance or for any other service, this may be a phishing scheme. Learn how to identify phishing emails and protect your accounts at https://explore.plos.org/phishing.

If we can help with anything else, please email us at customercare@plos.org.

Thank you for submitting your work to PLOS One and supporting open access.

Kind regards,

PLOS One Editorial Office Staff

on behalf of

Dr. Ramon Trullas

Academic Editor

PLOS One
